# Phylogenetic comparison of egg transparency in ascidians by hyperspectral imaging

**DOI:** 10.1038/s41598-020-77585-y

**Published:** 2020-11-30

**Authors:** Takumi T. Shito, Naohiro Hasegawa, Kotaro Oka, Kohji Hotta

**Affiliations:** 1 Department of Bioscience and Informatics, Faculty of Science and Technology, Keio University, Yokohama, 223-8522 Japan; 2 Department of Natural History Sciences, Graduate School of Science, Hokkaido University, Kita 10 Nishi 8 Kitaku, Sapporo, Hokkaido 060-0810 Japan; 3 Waseda Research Institute for Science and Engineering, Waseda University, 2-2 Wakamatsucho, Shinjuku, Tokyo, 162-8480 Japan; 4 Graduate Institute of Medicine, College of Medicine, Kaohsiung Medical University, Kaohsiung City, 80708 Taiwan

**Keywords:** Taxonomy, Evolutionary ecology

## Abstract

The transparency of animals is an important biological feature. Ascidian eggs have various degrees of transparency, but this characteristic has not yet been measured quantitatively and comprehensively. In this study, we established a method for evaluating the transparency of eggs to first characterize the transparency of ascidian eggs across different species and to infer a phylogenetic relationship among multiple taxa in the class Ascidiacea. We measured the transmittance of 199 eggs from 21 individuals using a hyperspectral camera. The spectrum of the visual range of wavelengths (400–760 nm) varied among individuals and we calculated each average transmittance of the visual range as bio-transparency. When combined with phylogenetic analysis based on the nuclear 18S rRNA and the mitochondrial cytochrome *c* oxidase subunit I gene sequences, the bio-transparencies of 13 species were derived from four different families: Ascidiidae, Cionidae, Pyuridae, and Styelidae. The bio-transparency varied 10–90% and likely evolved independently in each family. *Ascidiella aspersa* showed extremely high (88.0 ± 1.6%) bio-transparency in eggs that was maintained in the “invisible” larva. In addition, it was indicated that species of the Ascidiidae family may have a phylogenetic constraint of egg transparency.

## Introduction

Many marine organisms such as jellyfish, siphonophores, some crustaceans, pteropods, some squids, salps, and fish larvae are transparent^1–6^. Somewhat surprisingly, the biological role of transparency in living organisms appears to have received little attention presumably though it confers a selective advantage such as aiding approach to prey or avoidance of predators^2,4,7,8^. Even if an organism is transparent to predation, environmental UV radiation can still lead to pigmentation^9^. Therefore, the transparency of organisms can be affected and diversified by the surrounding environmental and ecological factors. Organismal transparency evolved independently multiple times in different animal phyla^10^. Still, little is known regarding how such transparency evolved within specific marine species. Some ascidians have transparent eggs/embryos^11,12^ and the oocytes of some styelids and pyurids are remarkable in having conspicuous cortical yellow to orange pigmented lipid droplets^13–15^, but the diversity of transparency in different ascidian eggs has not yet been investigated from a phylogenetic perspective.

Transparency depends on the transmission of incident light rather than absorption, scattering, or reflection^16^. Therefore, the measurement of transmittance should be used to evaluate this trait. The evaluation of transparency has been measured using a spectrophotometer and by specifying some wavelengths of the incident light^2^. In this case, transmittance is expressed as a percentage relative to sea water. Vertebrates detect light from 400 to 760 nm and this range was used here.

In this study, we precisely measured a wide range of the transmittance at 5-nm intervals via a hyperspectral camera and calculated the broad spectrum of visible transparency as “bio-transparency”. We present the first taxonomic exploration of bio-transparency in different ascidian eggs focused on the four different families in the orders Phlebobranchia and Stolidobranchia.

## Results

### Sampling of ascidians and identification

**Figure 1:**
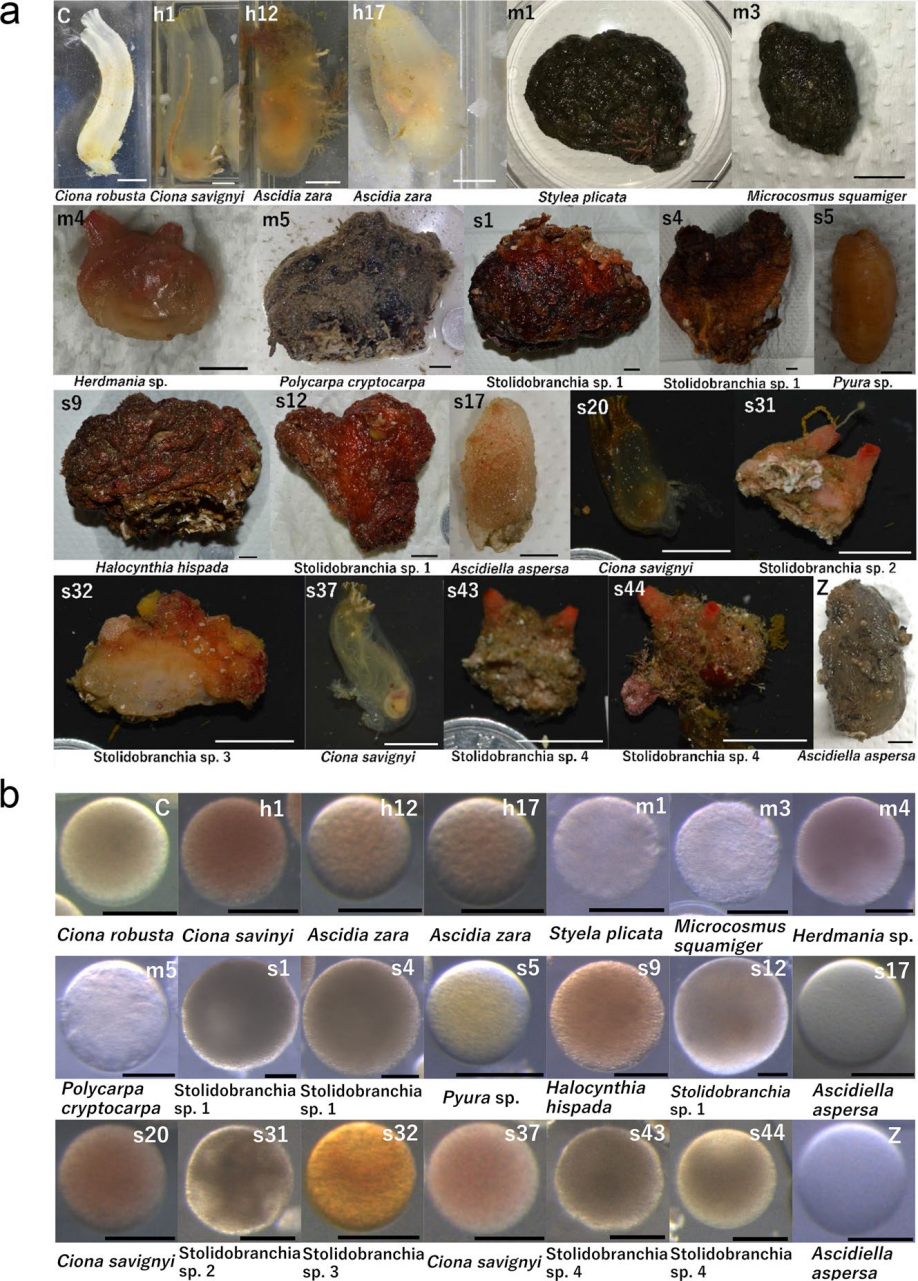
Pictures of ascidian samples used in this study. (**a**) Pictures of 21 sampled ascidians and (**b**) their isolated eggs (dechorionated). One individual C is a cultured *Ciona robusta* individual. Three individuals (h1, h12, and h17) are acquired at Honmoku. Four individuals (m1, m3, m4, and m5) are from Misaki, and 12 individuals (s1, s4, s5, s9, s12, s17, s20, s31, s32, s37, s43, and s44) are from Sado. One individual (Z) is from Onagawa. The scale bars of (**a**) shows 1 cm and (**b**) shows 100 µm.

To understand the diversity of transparency in different ascidian eggs, we collected wild solitary ascidians. A total of 99 individuals were randomly collected in four different locations: Honmoku, Misaki, Onagawa and Sado in Japan. Aquacultured *Ciona robusta* (*Ciona intestinalis* type A) was also added to our analysis. Individual IDs were initially assigned by combining the collection locations and serial numbers; for example, the individual obtained at Misaki sampled number 1 was called “m1” (Suppl. Table 1). A range of body sizes were observed (1.3–11 cm long). The smallest individual was s43 (Fig. 1a), and the largest was s1 (Fig. 1a). We identified the individuals based on anatomical features and comparisons between their molecular information and sequence data deposited in the DDBJ database. Some animals were identified at the species level (Suppl. Table 2) based on the nuclear 18S rRNA (18S) and mitochondrial cytochrome c oxidase subunit I (COI) gene sequences of each individual and deposited data (Suppl. Table 3). The individuals m1 and m3 were identified as *Styela plicata* and *Microcosmus squamiger*, respectively. The individuals m5 and s9 were identified as *Polycarpa cryptocarpa* and *Halocynthia hispida* with anatomical features. The individuals h12 and h17 were identified as *Ascidia zara*. The individuals s17 and Z had the most transparent eggs and were identified as *Ascidiella aspersa*^17^ and were sampled at two different locations. The individuals s20, s37, and h1 were the same species, *Ciona savignyi*. The individuals m4 and s5 were considered to be members of the genus *Herdmania* and *Pyura*, respectively, but we could not identify them at the species level. The sequences of s1, s4, and s12 were named as Stolidobranchia sp. 1 because they were entirely identical and regarded as the same species in the order Stolidobranchia but could not be identified at the family level. The individuals s31 and s32 were suspected to be different species in the order Stolidobranchia and named Stolidobranchia sp. 2 and sp. 3, respectively. The individuals s43, and s44 were regarded as the same species and named Stolidobranchia sp. 4, similar to Stolidobranchia sp. 1.

### Measurement of the egg transparency using hyperspectral camera

Ascidian eggs were isolated from 21 individuals (Fig. 1a). Of the 21 individuals, eggs with various colors and sizes were isolated (Fig. 1b). Egg diameters ranged from 120 μm (s5: *Pyura* sp.) to 381 μm (s12: Stolidobranchia sp. 1) (Fig. 1b; Suppl. Table 4).

**Figure 2:**
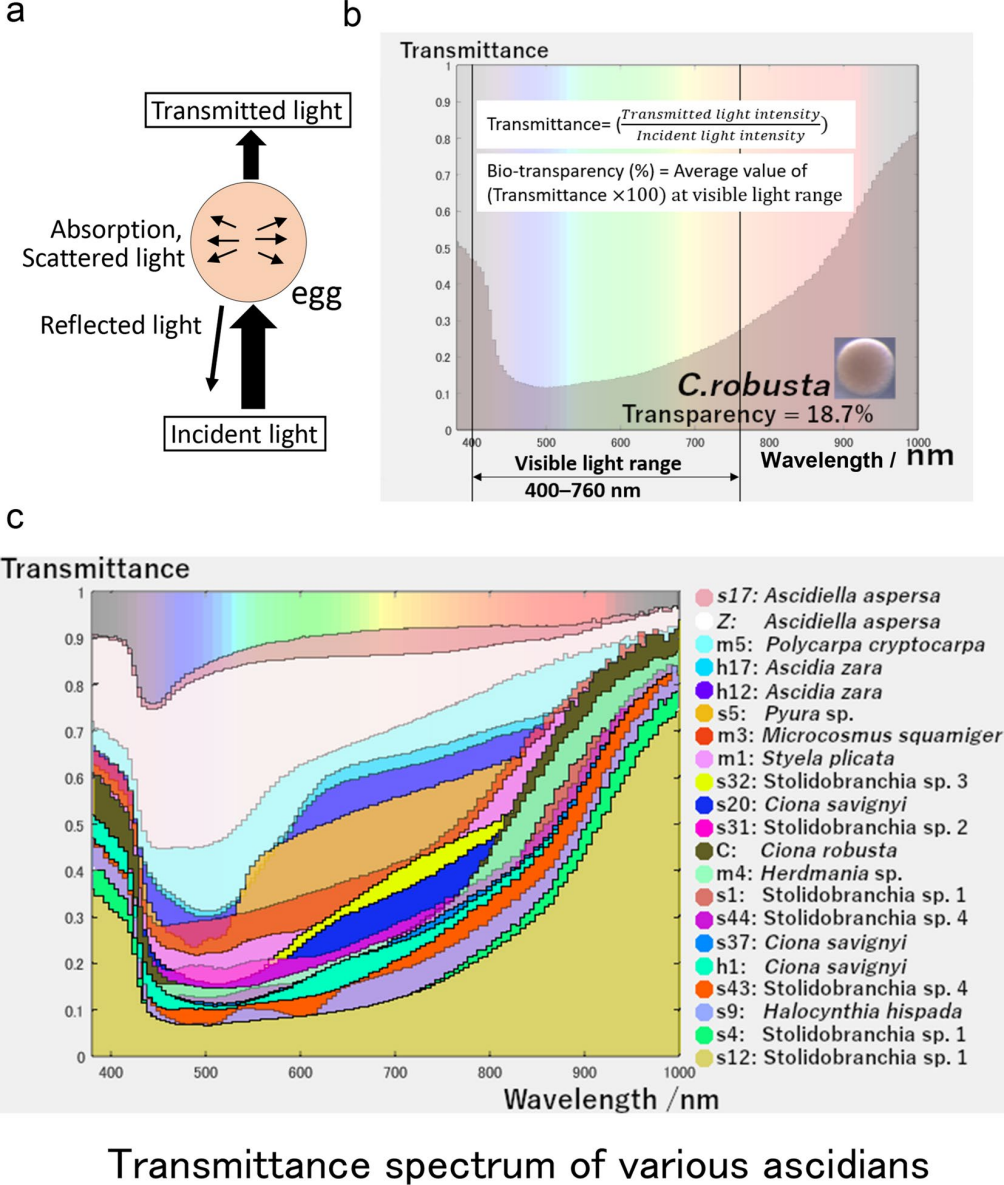
Measurement of ascidian egg transmittance. (**a**) The schematic relationships of the optic parameters, incident light, reflected light, absorption, scattered light, and transmitted light. When the incident light is applied to the egg, the intensity of the incident light decreases due to reflection and absorption and scattering when passing through the egg. The light is then detected as transmitted light. (**b**) The transmittance of *C. robusta* egg measured from 380 to 1000 nm by a hyperspectral camera. (**c**) The transmittance of eggs derived from 21 different individuals as measured by the hyperspectral camera.

Incident light is attenuated by reflection, scattering, and absorption; the light passes through the egg as transmitted light (Fig. 2a). Transmittance is the ratio of transmitted light to incident light (Fig. 2b). The hyperspectral camera could measure the transmittance over a wide wavelength range (380–1000 nm) simultaneously. The visible transmittance (440–760 nm) of eggs in *C. robusta* (Fig. 2b) was lower than the UV range (shorter than 400 nm) and IR range (above 800 nm). This trend was conserved in other individuals.

The spectra of the average transmittance of the eggs from the 21 individuals are shown in Fig. 2c. Overall, the UV range and IR range showed relatively higher transmittance in all eggs. On the other hand, the transmittance in the visible light range varied among individuals. For example, the transmittance of both m5 (*Polycarpa cryptocarpa*) and s5 (*Pyura* sp.) were reduced from 440 to 540 nm, whereas that of s9 (*Halocynthia hispada*) and s12 (Stolidobranchia sp. 1) were reduced over a broader range (440–700 nm; Fig. 2c; Suppl. Figure 1). Individual s9 (*Halocynthia hispada*) had two local minima at 500 nm and 600 nm. Individuals s17 and Z (*A. aspersa*) showed higher transmittance than the other individuals, maintaining high transmittance except for a small decrease from 440 to 540 nm (Suppl. Figure 1).

**Figure 3:**
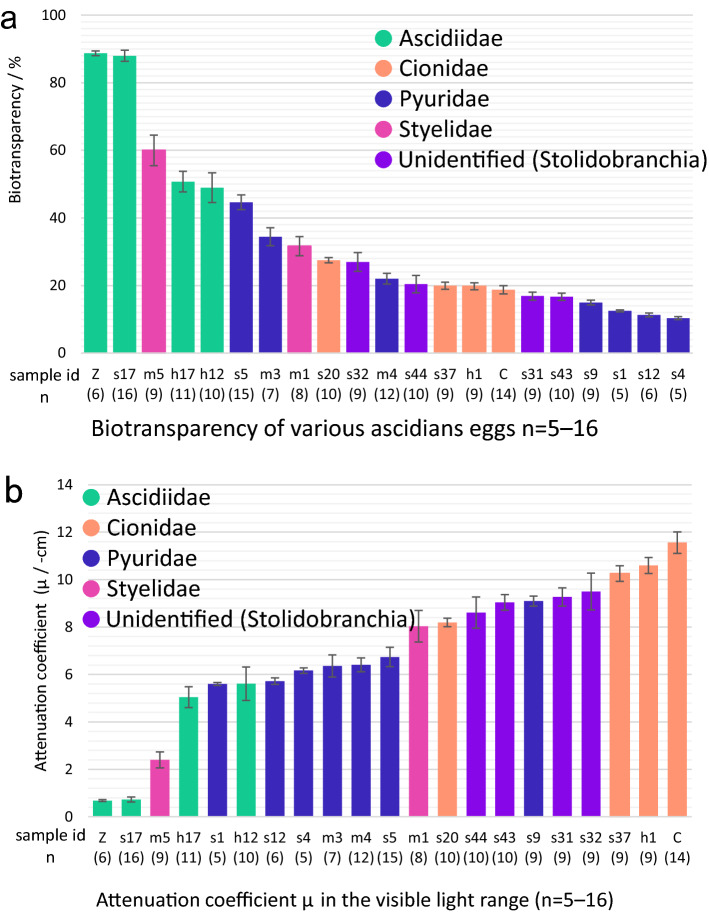
Bio-transparency and attenuation coefficient for different ascidian eggs. (**a**) Comparison of the bio-transparency of various ascidian eggs. (**b**) Attenuation coefficient in the visible light range of various ascidian eggs. The colors of the bars were divided into families or orders. The error bars indicate standard deviation.

To quantitatively measure the transparency of the visible light range, we next defined “bio-transparency” as the average value of transmittance at the visible light range 400–760 nm. The bio-transparency of *C. robusta* was 18.7% (Fig. 2b; Suppl. Figure 1) in the visible range. This is lower than other individuals. The highest bio-transparency was seen in Z and s17 (*A. aspersa*), 88.7 and 88.0%, respectively. The lowest bio-transparencies were in s4, s12, s1 (Stolidobranchia sp. 1), and s9 (*Halocynthia hispada*), but these values were not reflected in attenuation coefficients, which is the parameter that is considered the thickness of egg. A large attenuation coefficient means that the incident light is quickly "attenuated" (weakened) as it passes through the egg, and a small attenuation coefficient means that the egg is relatively transparent to the incident light. These two parameters are both useful estimating the transparency at the individual level (Fig. 3a) and the per unit length of the egg diameter (Fig. 3b), respectively. *Ascidiella aspersa* eggs (s17 and Z) are remarkably transparent in both parameters. For example, *C. robusta* was identified as the least transparent egg (Fig. 3b) and can be said to be opaque; however, in terms of bio-transparency, it is described as a model transparent organism^18–21^. Eggs of one unidentified Stolidobranchia species (s1, s4, and s12) showed low bio-transparency with a small attenuation coefficient because of the relatively large egg diameter (368 μm) (Suppl. Table 4). In contrast, individual s5 (*Pyura* sp.) had the smallest eggs and showed relatively high bio-transparency without a small attenuation coefficient.

### The relationship between egg transparency and phylogeny

**Figure 4:**
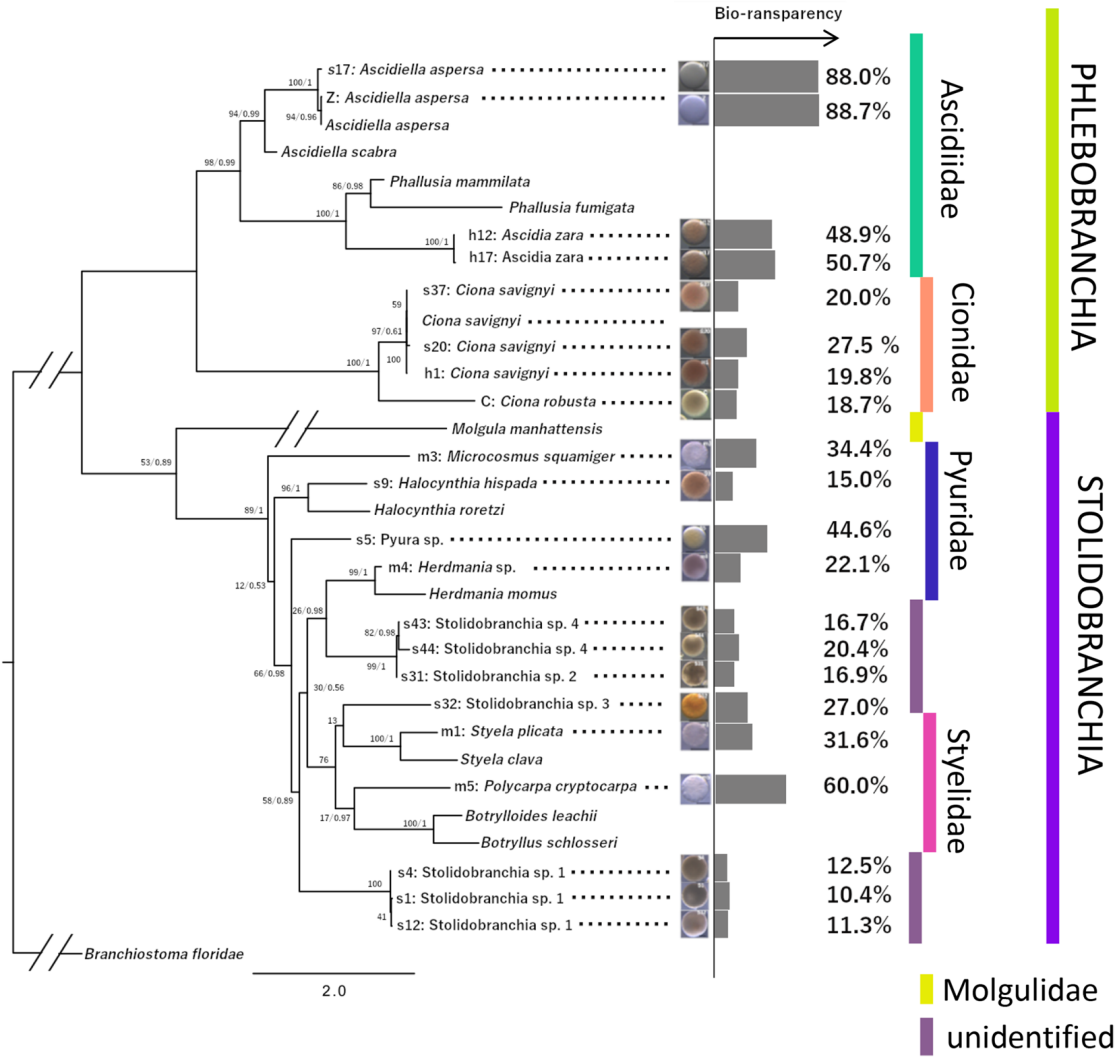
Phylogenetic relationships of 13 species in Ascidiacea and bio-transparency. The eggs of 13 species were shown by pictures. The ML tree was generated from concatenated sequences of COI and 18S. The numbers on the nodes indicate their bootstrap values. The individuals belong to one of the four families (green) belonging to the order Phlebobranchia or Stolidobranchia (blue).

**Figure 5:**
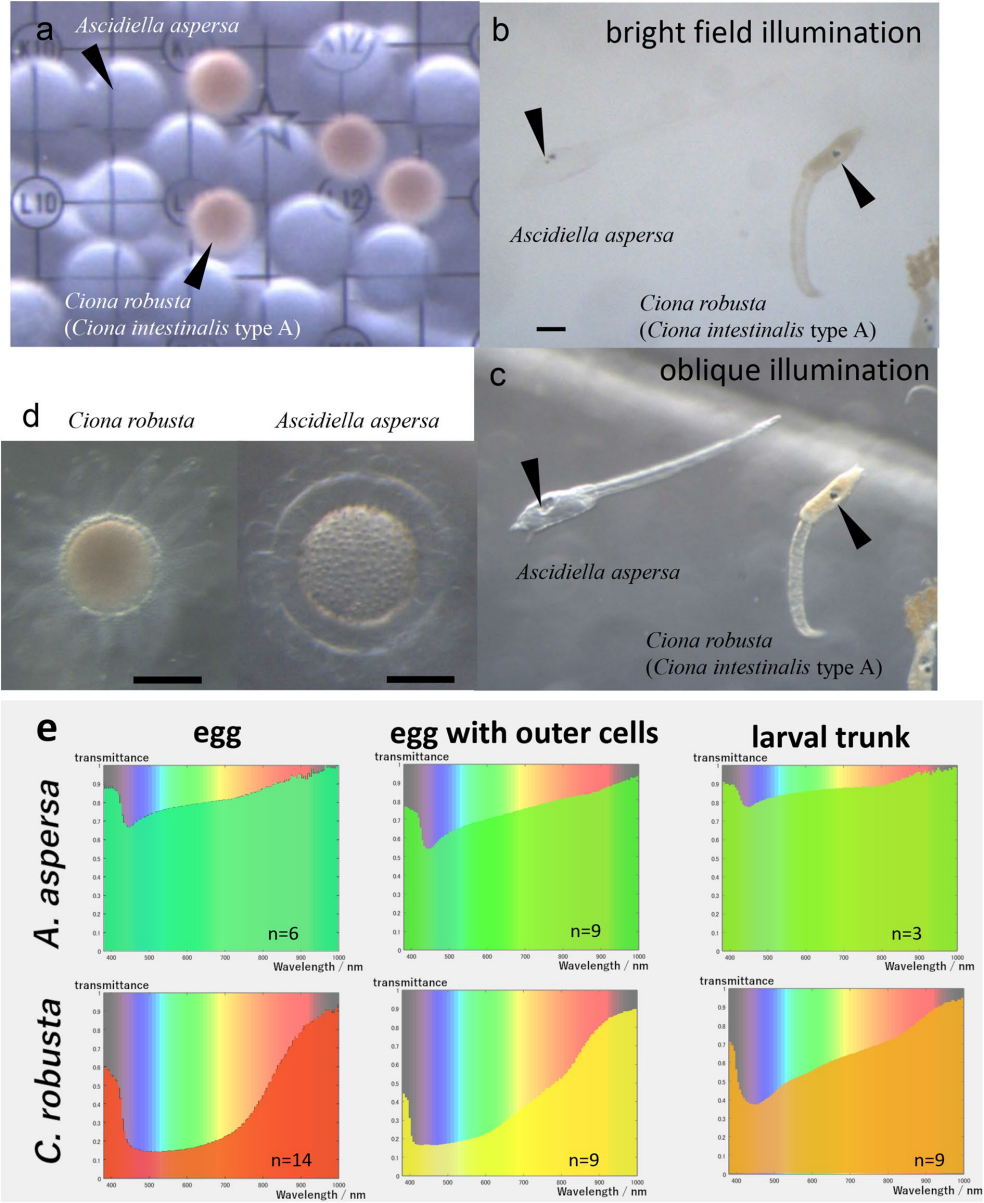
Conservation of the bio-transparency of egg and larva. (**a**) Comparison of the egg transparency between *Ascidiella aspersa* and *Ciona robusta*. (**b**) Bright field illumination of two larvae: *A. aspersa* and *C. robusta*. (**c**) Oblique illumination of two larvae showed in (**b**). The scale bar is 100 μm. (**d**) Pictures of eggs with outer cells (follicle cells and test cells) of *C. robusta* and *A. aspersa*. (**e**) The bio-transparency of egg, egg with outer cells and larval trunk in different ascidian species: *A. aspersa* and *C. robusta.*

Next, we investigated the egg bio-transparency from a phylogenetic perspective. The phylogenetic tree based on the sequence alignment of 18S and COI (Additional data 1) indicated that the 21 individuals derived from 13 different species in four families either in the order Phlebobranchia or Stolidobranchia (Fig. 4). The tree shape supports the more accurate phylogenetic tree obtained by transcriptomic data^22^. Based on the phylogenetic tree, ascidian egg transparency likely evolved independently in different families. For example, higher transmittance was observed in different families: s17 and Z (*A. aspersa* in Ascidiidae) and m5 (*Polycarpa cryptocarpa* in Styelidae). Interestingly, four of the top six most transparent eggs all belonged to the family Ascidiidae (Fig. 3, green bars; Suppl. Table 5, *A. aspersa* and *A. zara*).To test whether the bio-transparency of eggs were kept to later developmental stages, we next compared the transparency of eggs and larvae in both *A. aspersa,* which has transparent eggs, and *Ciona robusta,* which has relatively untransparent eggs (Fig. 5)*.* The bodies of *A. aspersa* larvae—except for pigment cells—are almost invisible under bright field illumination (Fig. 5a–c). The spectrum of transmittance in both eggs was comparable to that of both larvae (Fig. 5e); the eggs and larvae in *A. aspersa* had a higher transparency than *C. robusta*.

It has been described the extra-embryonic cells such as follicle cells and test cells are used to shield the embryo from potentially harmful UV-A and UV-B radiation^23^. Correspondingly, we found that eggs with outer cells in both species significantly reduced the shorter range of transparency (~ 400 nm) (Fig. 5e).

## Discussion

### The diversity of bio-transparency in ascidian eggs

We characterized the bio-transparency of ascidian eggs in each species by measuring the transmittance of a wide range of light using a hyperspectral camera. The results show that transmittance is not constant, especially in the visual wavelength range (Fig. 2c, Suppl. Figure 1), and the bio-transparency varied 10–90% among ascidian species (Fig. 3a), which could be one of the indicators of ascidian biodiversity (Fig. 2c, Suppl. Figure 1).

There may be several environmental and ecological factors that can affect egg transparency. Transparency can help organisms avoid visual predators^4^. Many marine animal larvae (coelenterates, siphonophores, many shrimplike crustaceans, gastropod mollusks, polychaete worms, salps, and fish) have highly transparent bodies^10,24^ providing them with almost perfect camouflage. Having transparent larval bodies may reduce the chances that large planktonic animals are eaten by visual predators^7,9^.

Interestingly, egg bio-transparency in *A. aspersa* is almost the same as larvae bio-transparency in this species (Fig. 5e). This means that high bio-transparency may take over from eggs to larvae. Some fish are known to be predators of ascidian larva^25^. The transparency in eggs and larvae may be beneficial for avoiding visual predators like other planktonic animals^10,24^. The adults used in this study are not transparent (Fig. 1a), so they may not use adult transparency for the avoidance of visual predation. In contrast to untransparent tunicates, some tunicates such as *Thetys vagina* (salp: Thaliacea) and *Rhopalaea* sp. have transparent tunics^5^ to avoid visual predation. On the other hand, solitary ascidians of the families Pyuridae and Styelidae may protect themselves from predators with thick, leathery tunics that are not transparent^26^. In this study, our hyperspectral imaging measured more than 380 nm. Since it is known that some fish have eyes that can detect UV^27^, relatively high transparency in the UV-A range (320–400 nm; Fig. 2c) might be a way these fish avoid predators with UV-vision.

UV-A (320–400 nm) can interfere significantly with embryological development^28–30^, and UV-A and UV-B (280–320 nm) can cause failure of normal ascidian development and larvae settlement^30^. It has been shown that ascidians have various strategies for UV protection in adults. The tropical species *Phallusia nigra* live in shallow waters that have more ambient light, including UV^31^. The black tunic of *Phallusia nigra* is considered to protect from UV^31^. *Didemnum mole* has mycosporine-like amino acids (MAAs)^32,33^. MAA absorb UV but transmit most of visible light range^32^. MAAs are found in the tunic of some ascidians^34,35^ and *Ascidia ceratode* eggs^23^. Since MAA cannot be synthesized in animals, the source of MAA could be the colonial tunic symbiont^35^ or the solitary ascidian food^23^. Although we could not measure the transparency of the full UV range in this study, it will be interesting to measure the transmittance of eggs in the UV range in future studies using multi-viewpoints.

The spectral diversification of the visible range in ascidian eggs (Fig. 2) may reflect different pigment content. It has been shown that some pigments, melanin and carotenoids are used as photoprotective compounds in zooplankton^36,37^. Although genetic heterogeneity in populations should be investigated^38^, Styela eggs contain pigmented cytoplasmic regions (see Fig. 4: m1: *Styela plicata*), which exhibit specific developmental fates^14^ including myoplasm, a yellow-pigmented cytoplasm and mRNAs^15^.

With the transparency of the ascidian egg and larvae, there may be a trade-off between reducing predation risk with a transparent body and protecting the body from UV with some pigments, such as those observed in zooplankton^9^. However, this trade-off may change due to changes in reproductive systems. In most colonial ascidians, eggs are brooded in the colony^13^, so egg transparency is less important for protection from predation. Relatively large eggs with large untransparent yolk granules^13^ may be due to changes in the reproductive system that could protect them from predators and enable them to store more yolk.

Among botryllid ascidians, some ovoviviparous species, such as *Botrylloides leachii* and *Botryllus schlosseri*, have dark, large, dense eggs very rich in yolk to sustain the long embryo development^39^. The phylogenetically close *Botrylloides violaceus*, which is viviparous, has a tiny, small amount of yolk and transparent eggs since the parent sustains the whole embryo development. This may have advantages over the more rapid development of yolkly eggs in terms of energy efficiency or the survivorship of larvae^40^. These examples show that the feature of “egg transparency” evolved within a clade (family or a genus) in relation to the reproductive strategy of the species^41^.

### High bio-transparency in the egg of Ascidiidae species

Our phylogenetic analysis suggested that the transparency of the eggs in two species of Ascidiidae, *A. zara* and *A. aspersa* is extremely high with a range of 48–88% (Figs. 3a, 4). Some other ascidiid ascidians (*Phallusia mammillata, Phallusia nigra* and *Ascidia ahodori*) have remarkably transparent eggs^11,42^ so egg transparency may be a common feature especially in this clade. Accordingly, we found that bio-transparencies in *A. aspersa* in different individuals within the same species only show 0.7% variance versus 7.2% variance in *Ciona savignyi* (the family Cionidae; Suppl. Table 5). These data suggest that the high bio-transparency is an evolutionarily conserved trait in the family Ascidiidae.

*Ascidiella asepersa* (s17, Z) had the highest bio-transparency among the species we collected (Fig. 3a, 88.0%, 88.7%). The bio-transparency was reduced, in part, with follicle cells and chorion (Fig. 5e), but remained relatively high. The high transparency is maintained during development at least from eggs to larvae (Fig. 5d). The bio-transparency of the eggs and larvae in *A. aspersa* may also play a role in the avoidance of the attack of predators. However, our data indicated that there is high transparency up to 400 nm range (Fig. 2) that may reflect the problem of the trade-off relationship involving the absence of UV protection by pigments and vulnerability to UV. Moreover, the follicle cells and chorion of *A. aspersa* eggs are highly buoyant; thus, the embryos reside at the air–water interface with potentially high UV exposure^23^.

*Ascidiella aspersa* is regarded as a notorious invasive species in the Global Invasive Species Database (2013). It is very interesting how *A. aspersa* protect UV damage. There are several possibilities suggested by Epel^23^: high titers of DNA repair enzymes that recover DNA from UV damage, high concentrations of egg-specific thiol compounds that are antioxidants, and developmental mechanisms that can accommodate DNA damage. The most likely possibility is some type of UV absorbance material. Ascidiid species *Ascidia ceratode* have MAAs in test cells and follicle cells and can absorb 90% of UV at 310 nm^23^. Interestingly, our data suggest that test cells and chorion reduce UV-A range (380–400 nm) transmittance of eggs (Fig. 5e). It is suggested that these outer cells play a very important role in protecting *A. aspersa* eggs against UV damage.

**Figure 6:**
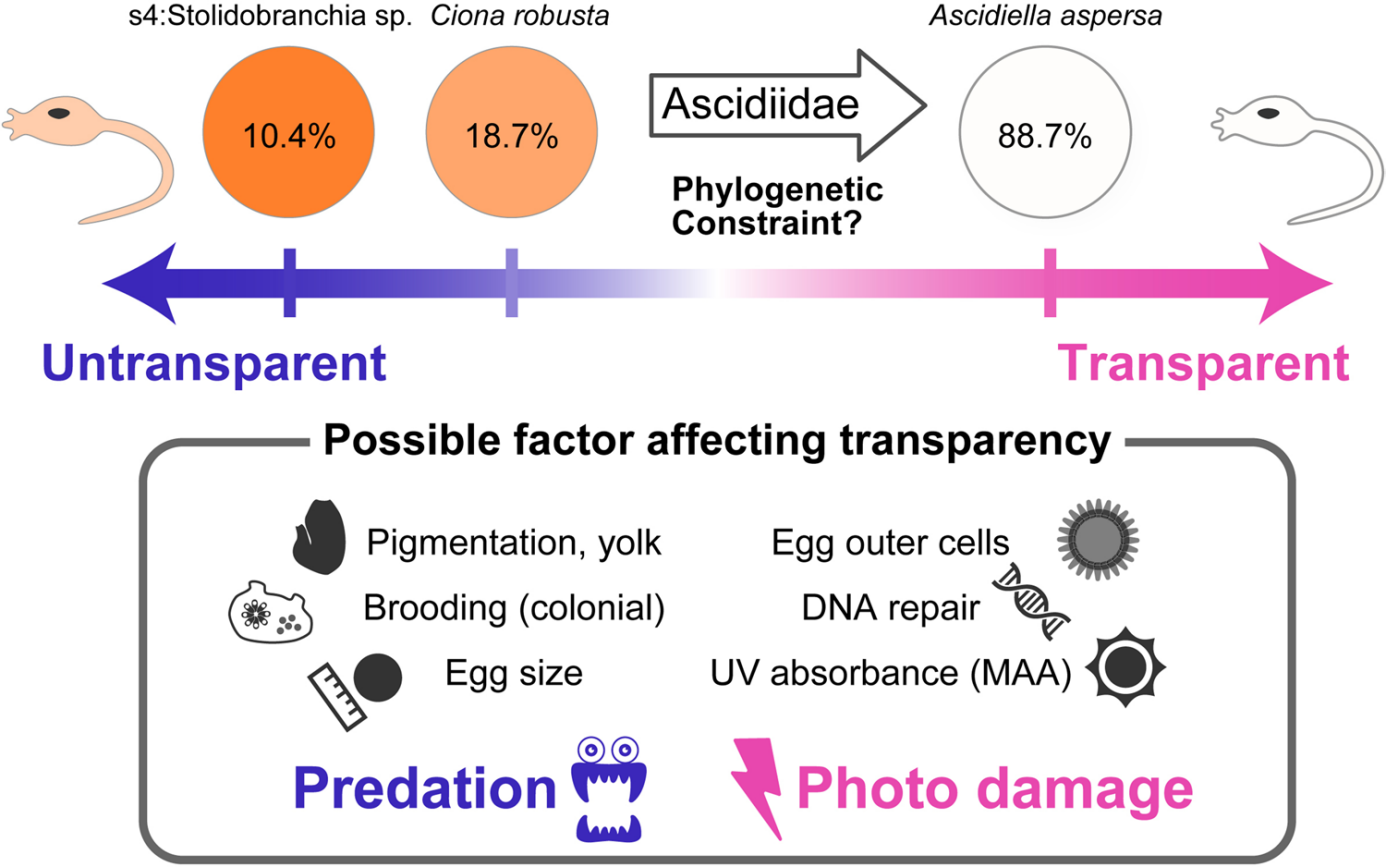
Conceptual diagram of a wide varieties of bio-transparency of ascidian eggs and factors that are thought to make the difference. Ascidians eggs have a wide variety of bio-transparencies (10–90%). There may be a trade-off relationship between reducing predation risk with a transparent body and protecting the body from UV with some pigments. Ascidiidae family may have a phylogenetic constraint of higher egg transparency. Various developmental and ecological factors possibly affect egg transparency. Higher transparency is considered to contribute to predator avoidance. Higher transmittance of the eggs is considered to provide less protection against photo damage. Possible affecting factors are pigmentation, yolk volume, changes in reproductive strategy to brooding in mother colony, and egg size, which allow the egg to become opaque. Other factors include protection by egg outer cells (follicle cells and test cells), DNA repair, and UV absorbance components (cf. MAAs), which allow the egg to become transparent.

Why were highly transparent eggs conserved in ascidiid ascidians during the evolutionary process? Given that egg transparency is considered to depend on multiple developmental and ecological factors, it clearly evolved independently in different taxa and can be very different in phylogenetically closed species. Therefore, a possible scenario is that the ancestral trait of egg transparency varied among individuals in different taxa, but the lineage of clade Ascidiidae then came under selective pressure (phylogenetic constraint) for higher transparency. Evidence to support this is that other ascidiid ascidians (*Phallusia mammillata, Phallusia nigra* and *Ascidia ahodori*) have remarkably transparent eggs^11,42^. As mentioned above, the spectrum of egg transparency may be the result of various developmental and ecological factors (Fig. 6).

### Mechanism of transparency

The transmittance spectrum across the visual range is different among ascidian species (Suppl. Figure 1) perhaps due to the presence of different colored yolk granules for each species. An organism or tissue is transparent if it neither absorbs nor scatters light^7^. The absence of colored yolk or pigments is not sufficient to make an object or organism transparent^3^. Although the morphological and physiological mechanisms in organismal transparency are poorly understood^4^, the important factors are a surface ultrastructure, a difference in refractive index from seawater, and a biochemical composition^3^. The ultrastructure of one salp tunic forms a nipple-like array that may produce an anti-reflective effect making this salp’s tunic barely visible in the water column^6^. Low-index lipids inserted into cell membranes may also help with transparency by matching the refractive index of the surrounding fluid^3^. The mechanism of ascidian transparency may also be elucidated by focusing on these factors in the future. The study of transparency is quite attractive because it contributes to various fields not only in ecological evolutionary developmental biology (eco-evo-devo) but also imaging systems and materials science^43^.

## Conclusion

We used a hyperspectral camera to measure the transparency of various Japanese ascidian eggs. We observed a wide variety of transparency values ranging from 10 to 90%, suggesting that egg transparency depends on multiple developmental and ecological factors. In addition, eggs of the Ascidiidae family were found to be particularly transparent.

## Methods

### Ascidian samples

A total of 99 ascidian individuals were collected at four places in Japan: Honmoku (Yokohama Honmoku Harbor, in Feb. 2020), Misaki (Misaki Marine Biological Station, University of Tokyo, in Aug. 2019), Onagawa (Onagawa Field Center, Tohoku University, in Oct. 2019), and Sado (Marine Biological Station, Sado Island Center for Ecological Sustainability, Niigata University, in Sep. 2019 and Mar. 2020). Gonoducts were dissected from live animals to sample the eggs; 21 different eggs were isolated. One individual *Ciona robusta* aquacultured at Misaki, three from Honmoku, four from Misaki, 12 from Sado, and one from Onagawa were tentatively named C, h1, h12, h17, m1, m3, m4, m5, s1, s4, s5, s9, s12, s17, s20, s31, s32, s37, s43, s44, and Z, respectively (Suppl. Table 1). The transparency of nearly all eggs was measured with a hyperspectral camera within a few hours whereas the eggs of individual m5 (*Polycarpa cryptocarpa)* were treated a few days later. A part of the gonad from each individual was stored in RNAlater (ThermoFisher, JAPAN) at − 30 °C until extraction of total RNA. The other body part was fixed in 99.5% EtOH and stored at room temperature for further identification. To compare the visual difference of the egg and larva in *Ciona robusta* and *A. aspersa*, both samples were imaged by oblique illumination mode and bright field illumination mode with Olympus SZX16 (Tokyo, Japan).

### Hyperspectral camera

Dechorionated egg samples in sea water were laid on plastic Petri dish at 20 °C, and the 380–1000 nm transmission spectra were measured at 5-nm intervals with a hyperspectral camera (EBA JAPAN, custom model NH-KO, Tokyo, Japan) on an inverted microscope (NIKON Eclipes IX71) with a 10X objective lens. Egg diameters were measured based on the image with imaging software GIMP version 2.10.18 (GPL license, free software). The bio-transparency was calculated at the ratio of the intensities between background and specimen using the hyperspectral camera.

### Calculating bio-transparency and attenuation coefficient

Transmittance (Τ) refers to the ratio between the intensity of incident light I_0_ on an object and the intensity of transmitted light I on the object

$$\begin{aligned} {\text{Transmittance}} & \\ & \tau = \frac{I}{{I_{0} }} \\ \end{aligned}$$

Bio-transparency was calculated as average value of (Τ × 100 at visible light range, 400–760 nm). When the thickness of the object is x, the attenuation coefficient μ is calculated using the following equation

$$\begin{aligned} {\text{Attenuation}}\;{\text{coefficient}} \\ & \mu = - \frac{1}{x}ln\frac{I}{{I_{0} }} \\ \end{aligned}$$

### Sequencing

Total RNAs were extracted from RNA later-preserved tissue samples using the RNeasy Mini kits (QIAGEN) or Direct-zol RNA Microprep R2061 (ZYMO RESEARCH) following the manufacturer’s protocols. The extracted total RNAs were reverse transcribed into cDNA libraries using Primescript II first strand cDNA synthesis kit (Takara Bio, Japan) following the manufacturer’s protocols.

The 18S gene was PCR-amplified using KOD One PCR Master Mix (TOYOBO, Japan) or CloneAmp HiFi PCR Premix (Takara Bio, Japan) either in two overlapping fragments of approximately 1 kb: 18S1/18S4 and 18S3/18S2. This step used the following primers: 18S1 (Fwd) 5′-CCTGGTTGATCCTGCCAG-3′, 18S2 (Rev) 5′-TAATGATCCATCTGCAGG-3′, 18S3 (Fwd) 5′-TTAGAGTGTTCAAAGCAGGC-3′, and 18S4 (Rev), adapted from the literature^44^.

The mitochondrial cytochrome *c* oxidase subunit I (COI) gene was PCR-amplified using KOD One PCR Master Mix (TOYOBO, Japan) or CloneAmp HiFi PCR Premix (Takara Bio, Japan) in two overlapping fragments of approximately 1 kbp. The following primer sets were used: LCO1490 (Fwd): 5′-GGTCAACAAATCATAAAGATATTGG-3′, HCO2198 (Rev): 5′-TAAACTTCAGGGTGACCAAAAAATCA-3′^45^.

PCR products were purified from 1.5% agarose gel using Wizard SV Gel Cleanup kit (Promega). The products were directly sequenced using BigDyeTerminator v3.1 Cycle Sequencing Kit (Applied Biosystems) on an ABI 310 sequencer. Base calling and assembly were performed with GeneStudio (TM) Professional Edition Version 2.2.0.0. Fouty new sequences were deposited in DDBJ database under Accession Numbers LC546997 to LC547016 (COI) and LC547313 to LC547331 (18S). In addition to these sequences, 12 other tunicate COI and 18S sequences were obtained from genbank (Suppl. Table 3).

### Phylogenetic analysis

Phylogenetic analyses were conducted using both Maximum Likelihood (ML) and Bayesian Inference (BI) approaches according to a previous study^46^. The 18S partial sequences were aligned with MAFFT ver.7 using the *E-INS-i* strategy^47^. Gblocks ver. 0.91b^48^ was used to eliminate poorly aligned positions and divergent regions in the 18S alignment. Partial sequences of COI were manually edited with MEGA7 ver. 7.0.26^49^. The sequences of 18S and COI were concatenated with MEGA7 ver. 7.0.26^49^. All ML analyses were performed using RAxML ver. 8.2.12^50^. Statistical support for the nodes was obtained by Bootstrap resampling with 1000 pseudo-replicates. Bayesian analyses were conducted using the programs MrBayes ver. 3.2.7a^51,52^. PartitionFinder ver. 2.1.1^53^ was used for selecting best-fit models of evolution for nucleotide. The program selected GTR + I + G model for one of three COI partitions and the GTR + G model for the other two partitions; GTR + I + G model was selected for 18S. Tracer ver. 1.7^54^ was used to assess run coverage and confirmed that the effective sample size of each parameter exceeded 200. In both phylogenetic analyses, *Branchiostoma floridae* was identified as an outgroup taxon.

## Supplementary information

Supplementary Information 1.

Supplementary Information 2.

## Data Availability

The datasets supporting the conclusions of this article are included in the article and its additional files.
