# Supplementary material for: Phylogenetic comparison of egg transparency in ascidians by hyperspectral imaging
Source: Sci Rep. 2020 Nov 30;10:20829. doi: 10.1038/s41598-020-77585-y (PMC7709464; doi:10.1038/s41598-020-77585-y)
Supplement: Supplementary file 1 — Supplementary Information 1. [file 41598_2020_77585_MOESM1_ESM.docx]

>m1

GCTTTTGTAATAATCTTTTTTTTCGTTATGCCCGTTATACTTAGGGGGTTTAGAAATTGGTTATTACCTCTCATGTTGAGGAGTCCAGACATAGCTTTTCCTCGATTAAATAATATAAGTTTTTGGCTATTACCGCCCGCGCTTGTTTTTTTGTTGATTAGTTCTTTTATTGAGAGTAGGGTGGGTACTAGATGGACGGTATATCCCCCACTAGCTGGAAACCTAGCCCACTCTGGGGGAGCACTAGATTGTGCCATTTTTTCCTTACATCTAGCTAGAATTTCTAGTATTTTAGGTTCTCTTAATTTTATGACTATGTTCAACATAAAAACTAAGTGGGTTATATTTTCAATACCTTTATTTTGTTGGACAGTCTTCTAGAGCTAATACATGCAAGGCGCGCTTTTATCAGTTTGATGACTCTGGATAACCACGCGGATCGTACGGTCTGACGTATCATTCAAGTGTCTGCCCTATCAACTGTCGAAGGTACGCTACTTGCCTACCTTGTGATAACGGGTAACGGGGAATCAGGGTTCGATTCCGGAGAGGGAGCCTGAGAAACGGCTACCACATCCAAGGAAGGCAGCAGGCGCGCAAATTACCCATTCCGACACGGGGAGGTAGTGACGAAAAATAACAAAACGAGGCCCTGTAATATGAGTACATCCTAAAACTCTTAACGAGTATCCATTGGAGGGCAAGTCTGGTGCCAGCAGCCGCGGTAATTCCAGCTCCAACAGTGTATGCTAAAGTTGTTGCGGTTGAAAAGCTCGTAGTTGGATTTTGGGCGAGCGCAGCCGGTCCGTCGCAGGGCGTGACTGGTTGCGTTCGCTTCGGTTCTCCGTCGGTGCTCTTGACTGAGTGTCGGCGGTGGCCGATAAGTTTACTTTGAAAAAATTAGAGTGTTCAAAGCAGGCTGCTCGCCTGCATAGTGTTGCATGGAATAATGGAATAGGACCTCGGTTCTATTTTGTTGGTTTTCGAGGTAATGATTAAGAGGGACGGGGGCGTCCGTTTCTTGGATCGGCGAACTACTGCGAAAGCATTCGCCAAGAATGTTTAATCAAGAGCGAAAGTCAGAGGTTCGAAGACGATCAGATACCGTCCTAGTTCTGACTATAAACGATGCCAACTAGCGATCGGGAGGCGTTACCATGACGACCTTTCCGGCAGGGGAAACCAAAGTCTTTGGGTTCCGGGGGAAGTATGGTTGCAAAGCTGAAACTTAAAGGAATTGACGGAAGGGCACCACCAGGAGTGGAGCCTGCGGCTTAATTTGACTCAACACGGGGAAACTCCGGCCCGGACACAGGTAGGATGACAGATTGAGAGCTCTTTCTTGATTCTGTG

>m3

GCTTTTGTAATAATCTTTTTTTTCGTTATGCCCATAATAATTAGGGGTTTTGGAAACTGGTTGTTACCTTTGATATTAGGATCTCCTGATATGGCTTTTCCACGGTTAAATAATATGAGTTTTTGGTTTTTACCCCCAGCATTTTTTCTACTTCTTATTTCTTCTCTTATTGAGAGCGGTGTTAGGACAGGTTGGACAGTTTACCCTCCTTTAGCTGGGAATTTAGCACATTCAAGGCCAGCATTAGATAGTGCTATTTTTTCACTACATTTGGCTAGGGTTTCTAGTATCTTGGGTAGTTTAAATTTTATAACTATATTTAATATAAAGGCAAAATGGAGATTATTTAATATACCTTTATTTGTATGGACTGTTTTCTAGAGCTAATaCaTgCGAGGCGTGCCTTTGTCAAGATGGcGAAAcTAGaCAaCCACGCGGTTCgCATGGTCTGAYGGATCATTCAAGTGTCTGCCCTATCAACTTTCGAAGGTACGTTACGTGCCTACCTTGTGATAACGGGTAACGGGGAATCAGGGTTCGATTCCGGAGAGGGAGCCTGAGAAACGGCTACCACATCCAAGGAAGGCAGCAGGCGCGCAAATTACCCATTCCGACACGGGGAGGTAGTGACGAAAAATAACAAAACGAGGCCCTGTAATATGAGCACAGTCTAAAGGTTTTAACGAGTATCCATTGGAGGGCAAGTCTGGTGCCAGCAGCCGCGGTAATTCCAGCTCCAACAGTGTATGCTAAAGTTGTTGCGGTTGAAAAGCTCGTAGTTGGATCTTGGCTGGGCGCCGCCGGTCCGTCGCAAGGCGTGACTGGCGGCGCTCTTTTCGGTTCGCCATCGGTGCTCTTGACTGAGTGTCGTCGGTGGCCGATGAGATTACTTTGAAAAAATTAGAGTGTTCAAAGCAGGCTCCTCGCCTGAATAGTGTTGCATGGAATAATGGAATAGGACCTCGGTTCTATTTTGTTGGTTTTTGAGGTAATGATTAAGAGGGACGGGGGCGTCCGTTTCTTGGATCGGCGAACTACTGCGAAAGCATTCGCCAAGAATGTTTAATCAAGAGCGAAAGTCAGAGGTTCGAAGACGATCAGATACCGTCCTAGTTCTGACTATAAACGATGCCAACTAGCGATCGGGAGGCGTTACCATGACGACCTTCCCGGCAGGGGAAACCAAAGTCTTTGGGTTCTGGGGGAAGTATGGTTGCAAAGCTGAAACTTAAAGGAATTGACGGAAGGGCACCACCAGGAGTGGAGCCTGCGGCTTAATTTGACTCAACACGGGGAAACTCCGGCCCGGACACAGGGAGGATGACAGATTGAGAGCTCTTTCTTGATTCTGTG

>m4

GCTTTTGTTATGATTTTTTTTTTTGTCATGCCCATTATAATTGGTGGGTTTGGGAACTGGCTATTACCTTTAATGGTAGGAAGTCCAGATATAGCGTTTCCTCGGATGAATAATATGAGTTTTTGGTTATTACCACCAGCTTTGTTCTTGTTGTTGATTAGTTCTGTGATTGAGGCTGGGGTTGGTACAGGGTGGACTGTCTATCCGCCGTTGGCTAGAAATTTAGCACATTCGGGTCCAGGTTTAGATTGTGCCATTTTTTCATTACATTTGGCTGGGGTTTCTAGTATTTTAGGGTCTTTGAATTTTATAACGTTGTTTAATATGAAGAGTAAGTGGGGTATATTTTCAATACCTTTATTTTGTTGAACTGTATTCTAGAGCTAATACATGCAAGGCGTGCTTTTATCAGTTTGATGACTCTGGATAACCACGCGGATCGCATGGTCTGACGAATCATTCAAGTGTCTGCCCTATCAACTTTCGAAGGTACGTTACGTGCCTACCTTGTGATAACGGGTGACGGGGAATCAGGGTTCGATTCCGGAGAGGGAGCCTGAGAAACGGCTACCACATCCAAGGAAGGCAGCAGGCGCGCAAATTACCCATTCCGACACGGGGAGGTAGTGACGAAAAATAACAAAACGAGGCTCTGTAATATGAGTACATTCTAAAACTCTTAACGAGTATCCATTGGAGGGCAAGTCTGGTGCCAGCAGCCGCGGTAATTCCAGCTCCAACAGTGTATGCTAAAGTTGTTGCGGTTGAAAAGCTCGTAGTTGGATCTTGGGTGGGTGTCGTCGGTCCGTCGCAAGGCGTGACTGGCGGCGCCTGCTTCGGTTCTTCGTCGGTGCTCTTGACTGAGTGTCGGCGGTGGCCGAAAAGTTTACTTTGAAAAAATTAGAGTGTTCAAAGCAGGCTGCTCGCCTGCATAGTGTTGCATGGAATAATGGAATAGGACCTCGGTTCTATTTTGTTGGTTTTCGAGGTAATGATTAAGAGGGACGGGGGCGTCCGTTTCTTGGATCGGCGAACTACTGCGAAAGCATTCGCCAAGAATGTTTAATCAAGAGCGAAAGTCAGAGGTTCGAAGACGATCAGATACCGTCCTAGTTCTGACTATAAACGATGCCAACTAGCGATCGGGAGGCGTTACCATGACGACCTTCCCGGCAGGGGAAACCAAAGTCTTTGGGTTCTGGGGGAAGTATGGTTGCAAAGCTGAAACTTAAAGGAATTGACGGAAGGGCACCACCAGGAGTGGAGCCTGCGGCTTAATTTGACTCAACACGGGGAAACTCCGGCCCGGACACAGGAAGGATGACAGATTGAGAGCTCTTTCTTGATTCTGTG

>m5

GCTTTTGTTATAATTTTTTTTTTTGTAATACCTATTATAATTAGGAGGTTCAGAAATTGGTTGCTTCCATTAATAGTTGGAAGTCCTGATATAGCTTTTCCTCGACTCAATAACATAAGTTTTTGATTACTGCCTCCAGCTTTATTTTTATTACTAATTTCATCAATAATTGAAAGTAGGGTTAGGACAGGTTGAACCGTGTACCCTCCGTTAGCTAGAAATCTAGCACACTCTAGAGCAGCTTTAGACTGTGCTATTTTTTCATTACACTTAGCAGGGGTATCAAGTATTCTGAGATCGTTAAATTTTATAACTTTGTTTAATATAAAGTCCAAGTGGGACATATTCTCCATACCACTTTTTTGTTGGACAGTATTCTAGAGCTAATACATGCGAGGCGTGCTTTTATCAGTTTGATGACTCTGGATAACCACGCGGATCGCACGGTCTGACATACCATTCAAGTGTCTGCCCTATCAACTTTCGAAGGTAGGCTACGGGCCTACCTTGTGATAACGGGTAACGGGGAATCAGGGTTCGATTCCGGAGAGGGAGCCTGAGAAACGGCTACCACATCCAAGGAAGGCAGCAGGCGCGCAAATTACCCATTCCGACACGGGGAGGTAGTGACGAAAAATAACAAAACGAGGCCCTGTAATATGAGTACATTCTAAAACTCTTAACGAGTATCCATTGGAGGGCAAGTCTGGTGCCAGCAGCCGCGGTAATTCCAGCTCCAACAGTATATGCTAAATTTGTTGCGGTTGAAAAGCTCGTAGTTGGACTTTGGGCGGGTGCAGCTGGTCCGTCGCAGGGCGTGACTGGCTGCGCTCGCTTCGGTTCTCCGTCGGTGCTCTTGACTGAGTGTCGGCGGTGGCCGAGAAGTTTACTTTGAAAAAATTAGAGTGTTCAAAGCAGGTTCGAATCCTGAATAGTGTTGCATGGAATAATGGAATAGGACCTCGGTTCTATTTTGTTGGTTTTCGAGGTAATGATTAAGAGGGACGGGGGCGTCCGTTTCTTGGATCGGCGAACTACTGCGAAAGCATTCGCCAAGAATGTTTAATCAAGAGCGAAAGTCAGAGGTTCGAAGACGATCAGATACCGTCCTAGTTCTGACTATAAACGATGCCAACTAGCGATCGGGAGGCGTTACCATGACGACCTTCCCGGCAGGGGAAACCAAAGTCTTTGGGTTCCGGGGGAAGTATGGTTGCAAAGCTGAAACTTAAAGGAATTGACGGAAGGGCACCACCAGGAGTGGAGCCTGCGGCTTAATTTGACTCAACACGGGGAAACTCCGGCCCGGACACAGGTAGGATGACAGATTGAGAGCTCTTTCTTGATTCTGTG

>S1

GCTTTTGTAATAATTTTTTTTTTTGTGATGCCTATTATAATTAGAAGGTTTGGAAATTGGTTATTGCCTTTAATGGTTGGGAGTCCTGATATGGCATTTCCTCGGATAAATAACATAAGTTTTTGGTTGTTACCTCCCGCATTGTTTTTGTTGTTAATTAGTTCTATAATTGAAAGTAGAGTAGGAACTAGGTGGACGGTATACCCTCCATTAGCTAGGAATTTAGCTCACTCGAGACCGAGGTTAGATGCGGCTATTTTTTCCTTACATATAGCGAGGGCTTCAAGTATTCTAAGGTCTATTAATTTTATAACTTTGTTTAATATAAAGGCTAAGTGGAATATATTTTCATTACCTTTATTTTGTTGAACAGTT------------------------------------------------------------------------------------------------------------------------------------------------------------------------------------------------------------------------------------------------------------------------------------------------------------------------------------------------------------------------------------------------------------------------------------------------------------------------------------------------------------------------------------------------------------------------------------------------------------------------------------------------------------------------------------------------------------------------------------------------------------------------------------------------------------------------------------------------------------------------------------------------------------------------------------------------------------------------------------------------------------------

>S4

GCTTTTGTAATAATTTTTTTTTTTGTGATGCCTATTATAATTAGAAGGTTTGGAAATTGGTTATTGCCTTTAATGGTTGGGAGTCCTGATATGGCATTTCCTCGGATAAATAACATAAGTTTTTGGTTGTTACCTCCTGCATTGTTTTTGTTGTTAATTAGTTCTATAATTGAAAGTAgAGTAGGAACTAGGTGGACGGTATACCCTCCATTAGCTAGGAATTTAGCTCATTCGAgACCGAGGTTAGATGCGGCTATTTTTTCCTTACACATAGCGAGGGCTTCAAGTATTCTAAGGTCTATTAATTTTATAACTTTGTTTAATATAAAGGCTAAGTGGAATATATTTTCATTACCTTTATTTTGTTGAACAGTTTTCTAGAGCTAATACATGCGAGGCGCGCTTTTATCAGTTTGACGACTCTGGGTAACCACGCGGATCGTATGGTCAGACGAACCATTCAAGTGTCTGCCCTATCAACTTTCGAAGGTACGTTACGCGCCTACCTTGTGATAACGGGTAACGGGGAATCAGGGTTCGATTCCGGAGAGGGAGCCTGAGAAACGGCTACCACATCCAAGGAAGGCAGCAGGCGCGCAAATTACCCATTCCGACCCGGGGAGGTAGTGACGAAAAATAACAAAACGAGGCCCTGTAATATGAGTACATTCTAAACCCTTTAACGAGTATCCATTGGAGGGCAAGTCTGGTGCCAGCAGCCGCGGTAATTCCAGCTCCAACAGTGTATGCTAAAGTTGTTGCGGTTGAAAAGCTCGTAGTTGGATCTTGGGTGGGCGCCGCCGGTCCGTCGCAAGGCGTGACTGGCGGCGCTCGCTTCGGTTCTCCGTCGGTGCTCTTGACTGAGTGTCGGCGGTGGCCGATAAGTTTACTTTGAAAAAATTAGAGTGTTCAAAGCAGGCCGTTTGCCTGCATAGTGTTGCATGGAATAATGGAATAGGACCTCGGTTCTATTTTGTTGGTTTTCGAGGTAATGATTAAGAGGGACGGGGGCGTCCGTTTCTTGGATCGGCGAACTACTGCGAAAGCATTCGCCAAGAATGTTTAATCAAGAGCGAAAGTCAGAGGTTCGAAGACGATCAGATACCGTCCTAGTTCTGACTATAAACGATGCCAACTAGCGATCGGGAGGCGTTACCATGACGACCTTCCCGGCAGGGGAAACCAAAGTCTTTGGGTTCTGGGGGAAGTATGGTTGCAAAGCTGAAACTTAAAGGAATTGACGGAAGGGCACCACCAGGAGTGGAGCCTGCGGCTTAATTTGACTCAACACGGGGAAACTCCGGCCCGGACACAGGTAGGATGACAGATTGAGAGCTCTTTCTTGATTCTGTG

>S5

GCGTTTGTTATAATTTTTTTTTTTGTTATACCTATTATAATTAGAGGATTTAGAAATTGATTATTACCATTAATATTAAGAAGTCCAGATATGGCTTTTCCTCGGTTAAATAACATAAGTTTTTGAATATTACCTCCTTCTTTGTTTTTGTTGCTTATTAGCTCTGTTATCGAAAGTAGAGTGAGAACTGGATGAACTGTTTATCCTCCTTTAGCTGGTAATTTAGCACATTCTGGTGCAGCCTTAGATTGTGCTATTTTTTCTTTACATCTTGCTGGTGTTTCAAGTATTTTAAGATCTTTAAATTTTATGACTATATTTAATATGAAAAGAAAATGAGGACTTTTTACAATACCTTTATTTTGTTGAACTGTTTTCTAGAGcTAATACATGCGAGGcGtGCTTTTATCAGTTTGAtGACTCTGGaTAACCACGCGGATCGTATGGTCtGACGAAtCATTCAAGTGTCTGCCCTATCAACTTTcGAAGGTACGTTACGtGCCTACCTTGTGATAACGGGTgACGGGGAATCAGGGTTCGATTCCGGAGAGGGAGCCTGAGAAACGGCTACCACATCCAAGGAAGGCAGCAGGCGCGCAAATTACCCATTCCGACaCGGGGAGGTAGTGACGAAAAATAACAAAACGAGGCCCTGTAATATGAGTACATTCTAAACCcTTTAACGAGTATCCATTGGAGGGCAAGTCTGGTGCCAGCAGCCGCGGTAATTCCAGCTCCAACAGTGTATGCTAAAGTTGTTGCGGTTGAAAAGCTCGTAGTTGGATCTTGGGTGGGCGCtGCCGGTCCGTCGCAAGGCGTGACTGGCcGCGCTCGCTTCGGTTCTCCGTCGGTGCTCTTGACTGAGTGTCGGCGGTGGCCGATAAGTTTACTTTGAAAAAATTAGAGTGTTCAAAGCAGGCtGcTcGCCTGaATAGTGTTGCATGGAATAATGGAATAGGACCTCGGTTCTATTTTGTTGGTTTTCGAGGTAATGATTAAGAGGGACGGGGGCGTCCGTTTCtTGGATCGGCGAACTACTGCGAAAGCATTCGCCAAGAATGTTTAATCAAGAGCGAAAGTCAGAGGTTCGAAGACGATCAGATACCGTCCTAGTTCTGACTATAAACGATGCCAACTAGCGATCGGGAGGCGTTACCATGACGACCTTCCCGGCAGGGGAAACCAAAGTCTTTGGGTTCTGGGGGAAGTATGGTTGCAAAGCTGAAACTTAAAGGAATTGACGGAAGGGCACCACCAGGAGTGGAGCCTGCGGCTTAATTTGACTCAACACGGGGAAACTCCGGCCCGGACACAGGTAGGATGACAGATTGAGAGCTCTTTCTTGATTCTGTG

>S9

GCTTTTTTAATAATTTTTTTTTTTGTGATACCTGTAATAATTAGGGGGTTTGGTAATTGGTTATTACCTTTGATATTAGGATGTCCAGATATGGCTTTTCCTCGTTTGAATAATATGAGTTTTTGGTTGTTGCCGCCATCGTTGTTTTTGTTGTTAGTTTCTTCTGTAACTGAGAGTGGGGTAAGGACTGGTTGAACAGTGTATCCTCCATTGGCTAGAAATTTGGCACATTCTGGGCCTGCTTTGGATTTTGCAATTTTTTCGTTACATTTGGCAGGTGTGTCTAGTATTTTAGGTGCCTTAAATTTTATAACTATGTTTAATATGAGGTTGAAGGGTGGAATGTTTCAAATAACGTTGTTTTGTTGGTCTGTATTCTAGAGCTAATACATGCGAGGCGTGCTTTTATCAGTTCGGTGACTCTGGATAACCGCGCGGATCGTATGGTCTGACGAACCATTCAAGTGTCTGCCCTATCAACTTTCGAAGGTACGTTACGTGCCTACCTTGTGATAACGGGTGACGGGGAATCGGGGTTCGATTCCGGAGAGGGAGCCTGAGAAACGGCTACCACATCCAAGGAAGGCAGCAGGCGCGCAAATTACCCATTCCGACACGGGGAGGTAGTGACGAAAAATAACAAAACGAGTCCCTGTAATATGAGAACATTCTAAACCCTTTAACGAGTATCCATTGGAGGGCAAGTCTGGTGCCAGCAGCCGCGGTAATTCCAGCTCCAACAGTGTATGCTAAAGTTGTTGCGGTTGAAAAGCTCGTAGTTGGATCTTGGGCGGGTGCAGCCGGTCCGTCGCAAGGCGTGACTGACTGCGCTCGCTTCGGTTCTCCGTCGGTGCTCTTGACTGAGTGTCGGCGGTGGCCGATAAGTTTACTTTGAAAAAATTAGAGTGTTCAAAGCAGGCTGCTCGCCTGAATAGTGTTGCATGGAATAATGGAATAGGACCTCGGTTCTATTTTGTTGGTTTTCGAGGTAATGATTAAGAGGGACGGGGGCGTCCGTTTCTTGGATCGGCGAACTACTGCGAAAGCATTCGCCAAGAATGTTTAATCAAGAGCGAAAGTCAGAGGTTCGAAGACGATCAGATACCGTCCTAGTTCTGACTATAAACGATGCCAACTAGCGATCGGGAGGCGTTACCATGACGACCTTCCCGGCAGGGGAAACCAAAGTCTTTCGGTTCCGGGGGAAGTATGGTTGCAAAGCTGAAACTTAAAGGAATTGACGGAAGGGCACCACCAGGAGTGGAGCCTGCGGCTTAATTTGACTCAACACGGGGAAACTCCGGCCCGGACACAGGTAGGATGACAGATTGAGAGCTCTTTCTTGATTCTGTG

>S12

GCTTTTGTAATAATTTTTTTTTTTGTGATGCCTATTATAATTAGAAGGTTTGGAAATTGGTTATTGCCTCTAATGGTTGGGAGTCCTGATATGGCATTTCCTCGGATAAATAACATAAGTTTTTGGTTGTTACCTCCTGCATTGTTTTTGTTGTTAATTAGTTCTATAATTGAAAGTAGAGTAGGAACTAGGTGGACGGTATACCCTCCATTAGCTAGGAATTTAGCTCATTCGAGACCGAGGTTAGATGCGGCTATTTTTTCCTTACATATAGCGAGGGCTTCAAGTATTCTAAGGTCTATTAATTTTATAACTTTGTTTAATATAAAGGCTAAGTGGAATATATTTTCATTACCTTTATTTTGTTGAACAGTTTTCTAGAGCTAATACATGCGGGGCGCGCTTTTATCAGTTTGACGACTCTGGGTAACCACGCGGATCGTATGGTCAGACGAACCATTCAAGTGTCTGCCCTATCAACTTTCGAAGGTACGTTACGCGCCTACCTTGTGATAACGGGTAACGGGGAATCAGGGTTCGATTCCGGAGAGGGAGCCTGAGAAACGGCTACCACATCCAAGGAAGGCAGCAGGCGCGCAAATTACCCATTCCGACCCGGGGAGGTAGTGACGAAAAATAACAAAACGAGGCCCTGTAATATGAGTACATTCTAAACCCTTTAACGAGTATCCATTGGAGGGCAAGTCTGGTGCCAGCAGCCGCGGTAATTCCAGCTCCAACAGTGTATGCTAAAGTTGTTGCGGTTGAAAAGCTCGTAGTTGGATCTTGGGTGGGCGCCGCCGGTCCGTCGCAAGGCGTGACTGGCGGCGCTCGCTTCGGTTCTCCGTCGGTGCTCTTGACTGAGTGTCGGCGGTGGCCGATAAGTTTACTTTGAAAAAATTAGAGTGTTCAAAGCAGGCCGTTTGCCTGCATAGTGTTGCATGGAATAATGGAATAGGACCTCGGTTCTATTTTGTTGGTTTTCGAGGTAATGATTAAGAGGGACGGGGGCGTCCGTTTCTTGGATCGGCGAACTACTGCGAAAGCATTCGCCAAGAATGTTTAATCAAGAGCGAAAGTCAGAGGTTCGAAGACGATCAGATACCGTCCTAGTTCTGACTATAAACGATGCCAACTAGCGATCGGGAGGCGTTACCATGACGACCTTCCCGGCAGGGGAAACCAAAGTCTTTGGGTTCTGGGGGAAGTATGGTTGCAAAGCTGAAACTTAAAGGAATTGACGGAAGGGCACCACCAGGAGTGGAGCCTGCGGCTTAATTTGACTCAACACGGGGAAACTCCGGCCCGGACACAGGTAGGATGACAGATTGAGAGCTCTTTCTTGATTCTGTG

>S17

GCTTTTGTTATGATTTTTTTTTTTGTTATGCCTATTATAATTGGCGGGTTTGGTAATTGGCTGTTGCCTATAATAGTTGGTGCTCCAGATATGGCTTTTCCTCGTATGAATAATATGAGTTTTTGACTACTTCCACCTGCATTTTTTATGTTGATTATTTCTTCCATGATTAGGACTGGGGCGGGGACGGGGTGGACAGTGTATCCCCCTCTCTCAGGGAACTTGGCCCATGCGGGCCCGGCTGTTGATTGTGCAATTTTTTCTTTACACTTGGCTGGTGTTTCTAGTATTCTCGGTTCTATTAATTTCTTAGTGATAATGGGTATGAAGGCGGGGTTTAGATATTTAAACTTGAGTTTGTTTTGCTGGTCTGTATGCTAGAGCTAATACATGCGAGGCGTGCTTTTATCAGTTTGGTGACTCTGGATAACTTTGCGGATCGCACGGTCTGACGGATCATTCAAGTGTCTGCCCTATCAACTTTCGTCGGTACGGTACTGGCCTACCGAGTTCTTACGGGTAACGGAGAATCAGGGTTCGATTCCGGAGAGGGAGCCTGAGAAACGGCTACCACATCCAAGGAAGGCAGCAGGCGCGCAAATTACCCATTCCGACGCGGGGAGGTAGTGACGAAAAATAACAAAACGAGGCCCTGTAATATGAGTACACTTTAAAACCTTTAACGAGTATCCATTGGAGGGCAAGTCTGGTGCCAGCAGCCGCGGTAATTCCAGCTCCAAAAGTGTATATTTAAGTTGTTGCGGTTAAAAAGCTCGTAGTTGGATTTTGGGCGCAGGCGGCCGGTCCGTCGCAAGGCGTGACTGGCCGTCCTGGCTCCGGTTCTCCGCCGGTGCCCTTGACTGAGTGTCGGCGGCGGCCGGAACGTTTACTTTGAAAAAATTAGAGTGTTCAAAGCAGGCGATTCGCCTGAATAATGGTGCATGGAATAATGGAATAGGACCTCGGTTCTATTTTGTTGGTTTTCGAGGTAATGATTAAGAGGGACGGGGGCATTCGTTTCTTGGATCGGCGAACGACTGCGAAAGCATTTGCCAAGAATGTTTAATCAAGAGCGAAAGTCAGAGGTTCGAAGACGATCAGATACCGTCCTAGTTCTGACTATAAACGATGCCAACTAGCGATCGGGGGGCGTTAGTTTTACGACCTCTCCGGCAGGGGAAACCAAAGTCTTTGGGTTCCGGGGGAAGTATGGTTGCAAAGCTGAAACTTAAAGGAATTGACGGAAGGGCACCaCCAGGAGTGGAGCCTGCGGCTTAATtTGACTCAACACGGGAAATCTCCGGCCCGGACACAGTGAGGATGACAGATTGAGAGCTCTTTCTTGATTCTGTG

>Z

GCTTTTGTTAtGATTTTTTTTTTTGTTATGCCTATTATAATTGGCGGGTTTGGTAATTGGTTGTTGCCTATAATAGTTGGTGCTCCAgATATGGCTTTTCCTCGTATGAATAATATGAGTTTTTGACTACTTCCaCCTGCATTTTTTATATTGATTATTTCTTCCATGATTAGGACTAGGGCTGGGACGGGATGGACAGTGTATCCCCCTCTCTCAGGtAACTTAgCTCATGCGGGCCCGGCTGTTGATTGTGCAATTTTTTCTTTACACCTGGCTGGtGTTTCTAGTATTCTCGGTTCTATTAATTTCTTAGTGATGATAGGTATGAAGGCGGGGTTTAGTTATTTAAACTTAAGTTTATTTTGCTGGTCTGTATGCTAGAGCTAATACATGCGAGGCGTGCTTTTATCAGTTTGGTGACTCTGGATAACTTTGCGGATCGCACGGTCTGACGGATCATTCAAGTGTCTGCCCTATCAACTTTCGTCGGTACGGTACTGGCCTACCGAGTTCTTACGGGTAACGGAGAATCAGGGTTCGATTCCGGAGAGGGAGCCTGAGAAACGGCTACCACATCCAAGGAAGGCAGCAGGCGCGCAAATTACCCATTCCGACGCGGGGAGGTAGTGACGAAAAATAACAAAACGAGGCCCTGTAATATGAGTACACTTTAAAACCTTTAACGAGTATCCATTGGAGGGCAAGTCTGGTGCCAGCAGCCGCGGTAATTCCAGCTCCAAAAGTGTATATTTAAGTTGTTGCGGTTAAAAAGCTCGTAGTTGGATTTTGGGCGCAGGCGGCCGGTCCGTCGCAAGGCGTGACTGGCCGTCCTGGCTCCGGTTCTCCGCCGGTGCCCTTGACTGAGTGTCGGCGGCGGCCGGAACGTTTACTTTGAAAAAATTAGAGTGTTCAAAGCAGGCGATTCGCCTGAATAATGGTGCATGGAATAATGGAATAGGACCTCGGTTCTATTTTGTTGGTTTTCGAGGTAATGATTAAGAGGGACGGGGGCATTCGTTTCTTGGATCGGCGAACGACTGCGAAAGCATTTGCCAAGAATGTTTAATCAAGAGCGAAAGTCAGAGGTTCGAAGACGATCAGATACCGTCCTAGTTCTGACTATAAACGATGCCAACTAGCGATCGGGGGGCGTTAGTTTTACGACCTCTCCGGCAGGGGAAACCAAAGTCTTTGGGTTCCGGGGGAAGTATGGTTGCAAAGCTGAAACTTAAAGGAATTGACGGAAGGGCACCACCAGGAGTGGAGCCTGCGGCTTAATTTGACTCAACACGGGAAATCTCCGGCCCGGACACAGTGAGGATGACAGATTGAGAGCTCTTTCTTGATTCTGTG

>Styela_clava

GCCTTCGTAATGATTTTTTTTTTTGTTATGCCGGTAATAATTAGAAGATTTAGGAATTGATTGTTACCTTTGATGTTGGGAAGTCCGGACATAGCTTTTCCACGTCTAAATAATATGAGTTTTTGGTTATTGCCTCCGGCGGTAGTCCTTTTATTAGTAAGCTCCTTTATTGAGAGTGGAGTAGGAACTAGGTGGACTGTGTACCCTCCTTTAGCTAGGAACTTGGCCCATTCAAGAGGTGCTCTTGATTGTGCTATTTTTTCTTTACATTTGGCGGGGATCTCCAGTATTTTAAGATCTCTTAATTTTATAACTATATTTAATATAAAGACAAAATGAGTAATATTTTCTATGCCATTATTTTGTTGGACTGTATTCTAGAGCTAATACATGCAAGGCGCGCTTTTATCAGTTTGATGACTCTGGATAACCACGCGGATCGTACGGTCTGACGTATCATTCAAGTGTCTGCCCTATCAACTGTCGAAGGTACGCTACGTGCCTACCTTGTGATAACGGGTAACGGGGAATCAGGGTTCGATTCCGGAGAGGGAGCCTGAGAAACGGCTACCACATCCAAGGAAGGCAGCAGGCGCGCAAATTACCCATTCCGACACGGGGAGGTAGTGACGAAAAATAACAAAACGAGGCCCTGTAATATGAGTACATCCTAAAACTCTTAACGAGTATCCATTGGAGGGCAAGTCTGGTGCCAGCAGCCGCGGTAATTCCAGCTCCAACAGTGTATGCTAAAGTTGTTGCGGTTGAAAAGCTCGTAGTTGGATTTTGGGCGAGCGCCGCCGGTCCGTCGCAAGGCGTGACTGGTTGCGTTCGCTTCGGTTCTCCGTCGGTGCTCTTGACTGAGTGTCGGCGGTGGCCGATAAGTTTACTTTGAAAAAATTAGAGTGTTCAAAGCAGGCTGTTCGCCTGCATAGTGTTGCATGGAATAATGGAATAGGACCTCGGTTCTATTTTGTTGGTTTTCGAGGTAATGATTAAGAGGGACGGGGGCGTCCGTTTCTTGGATCGGCGAACTACTGCGAAAGCATTCGCCAAGAATGTTTAATCAAGAGCGAAAGTCAGAGGTTCGAAGACGATCAGATACCGTCCTAGTTCTGACTATAAACGATGCCAACTAGCGATCGGGAGGCGTTACCATGACGACCTTTCCGGCAGGGGAAACCAAAGTCTTTGGGTTCCGGGGGAAGTATGGTTGCAAAGCTGAAACTTAAAGGAATTGACGGTAGGGCACCACCAGGAGTGGAGCCTGCGGCTTAATTTGACTCAACACGGGGAAACTCCGGCCCGGACACAGGTAGGATGACAGATTGAGAGCTCTTTCTTGATTCTGTG

>Halocynthia_roretzi

GCCTTTGTTATAATTTTCTTTTTTGTTATGCCGGTAATAATTGGTAGATTTAGAAATTGATTGTTACCTTTGATATTAGGATGTCCAGATATGGCTTTTCCTCGATTAAATAATATGAGTTTTTGGTTATTGCCTCCTGCGTTATTTATGCTATTGTTATCTTCAGTAATTGAGAGTGGTGTAAGAACAGGTTGGACTGTTTATCCACCTTTATCAAGGAATTTAGCTCATTCTGGGCCGGCACTTGATTGTGCTATTTTTTCTTTGCATTTAGCTAGAGTGTCTAGTATTTTAGGTTCTTTGAATTTTATAACTATATTTAATATGGGTTTAAAGGGTGGTTTATTTATAATACCATTATTTTGTTGGTCTGTGTTCTAGAGCTAATACATGCGAGGCGTGCTTTTATCAGTTTGATGACTCTGGATAACCACGCGGATCGTATGGTCTGACGAATCATTCAAGTGTCTGCCCTATCAACTTTCGAAGGTAGCTTACGTGCCTACCTTGTGATAACGGGTGACGGAGAATCAGGGTTTGATTCCGGAGAGGGAGCCTGAGAAACGGCTACCACATCCAAGGAAGGCAGCAGGCGGGCAAATTACCCATTCCGACACGGGGAGGTAGTGACGAAAAATAACAAAACGAGTCCCTGTAATATGAGTACATTCTAAACCCTTTAACGAGTATCCATTGGAGGGCAAGTCTGGTGCCAGCAGCCGCGGTAATTCCAGCTCCAACAGTGTATGCTAAAGTTGTTGCGGTTGAAAAGCTCGTAGTTGGATCTTGGGTGGGTGCAGCCGGTCCGTCGCAAGGCGTGACTGGCTGCGCTCGCTTCGGTTCTCCGTCGGTGCTCTTGACTGAGTGTCGGCGGTGGCCGATAAGTTTACTTTGAAAAAATTAGAGTGTTCAAAGCAGGCTGCTCGCCTGAATAGTGTTGCATGGAATAATGGAATAGGACCTCGGTTCTATTTTGTTGGTTTTCGAGGTAATGATTAAGAGGGACGGGGGCGTCCGTTTCTTGGATCGGCGAACTACTGCGAAAGCATTCGCCAAGAATGTTTAATCAAGAGCGAAAGTCAGAGGTTCGAAGACGATCAGATACCGTCCTAGTTCTGACTATAAACGATGCCAACTAGCGATCGGGAGGCGTTACCATGACGACCTTCCCGGCAGGGGAAACCAAAGTCTTTCGGTTCCGGGGGAAGTATGGTTGCAAAGCTGAAACTTAAAGGAATTGACGGAAGGGCACCACCAGGAGTGGAGCCTGCGGCTTAATTTGACTCAACACGGGGAAACTCCGGCCCGGACACAGGTAGGATGACAGATTGAGAGCTCTTTCTTGATTCTGTG

>Ascidiella_scabra

GCTTTTGTTATGATTTTTTTCTTTGTTATGCCCATTATGATTGGTGGGTTTGGTAATTGGTTGTTACCTATGATAGTAGGGGCTCCGGATATGGCTTTCCCTCGTATGAATAATATGAGTTTTTGGTTACTCCCTCCGGCATTTTTTATGTTGATTATTTCTTCTGTTATTGGGAGTGGAGCAGGTACGGGGTGGACAGTATATCCACCGCTTTCTAGCAATCTAGCCCACGCGGGCCCTGCTGTTGATTGTGCTATTTTTTCTTTACACCTAGCCAGAGTTTCAAGTATTCTTGGGTCTATTAATTTTTTAGTAATAATAGGCATGAAGGCTAAGTTCAGTTACTTGAATTTGAGTTTATTTTGTTGGTCTGTT------------------------------------------------------------------------------------------------------------------------------------------------------------------------------------------------------------------------------------------------------------------------------------------------------------------------------------------------------------------------------------------------------------------------------------------------------------------------------------------------------------------------------------------------------------------------------------------------------------------------------------------------------------------------------------------------------------------------------------------------------------------------------------------------------------------------------------------------------------------------------------------------------------------------------------------------------------------------------------------------------------------

>Phallusia_mammillata

GCCTTTGTGATAATCTTCTTTTTCGTCATGCCCATTATAATCGGCGGGTTTGGTAATTGGCTTTTGCCTATGATAGTGGGGGCGCCTGACATGGCATTCCCCCGTCTTAATAATATGAGCTTCTGGCTACTTCCTCCGGCTTTATTTCTTCTACTAGTCTCTTCCGTGGTTGGGAGTGGGGCCAGAACAGGTTGGACTGTGTACCCCCCTCTTGCTGGCAATTTAGCTCATGCGGGTGCCTCCGTGGATTGTGCTATTTTTTCCCTCCACTTAGCGGGGGTTTCCAGTATTCTCGGGTCGATTAATTTTCTTGTAATGATGAACATAAAGGCTAAGTTTGGGGCCTATAATATTAGTCTTTTTTGTTGGTCGGTAATCTAGAGCTAATACATGCGGGGCGTGCATTTATCAGCTTGGCGACTCTGGGTAACTCAGCGGATCGCACGGTCTGACGGATCATTCAAGTGTCTGCCCTATCAACTTTCGTCGGTAGGGTATCGGCCTACCGAGTTCTTACGGGTGACGGGGAATCAGGGTTCGATTCCGGAGAGGGAGCCTGAGAAACGGCTACCACATCCAAGGAAGGCAGCAGGCGCGCAAATTACCCATTCCGACGCGGGGAGGTAGTGACGAAAAATAACAAAACGAGGCCCTGTAATATGAGCACACTTTAAAACCTTTAACGAGTATCCATTGGAGGGCAAGTCTGGTGCCAGCAGCCGCGGTAATTCCAGCTCCAAAAGTGTATATTTAAGTTGTTGCGGTTAAAAAGCTCGTAGTTGGATCTTGGGCGCAGGCGGCCGGTCCGTCGCAAGGCGTGACTGGCCGCACCTGCCCCGGCGCTCCGCCGGTGCCCTTGACTGAGTGTCGGCGGCGGCCGGGACGTTTACTTTGAAAAAATTAGAGTGTTCAAAGCAGGCGGCTCGCCTGAATAATGGTGCATGGAATAATGGAATAGGACCTCGGTTCTATTTTGTTGGTTTTCGAGGTAATGATTAAGAGGGACGGGGGCATTCGTTTCTTGGATCGGCGAGCGACTGCGAAAGCATTTGCCAAGAATGTTTAATCAAGAGCGAAAGTCAGAGGTTCGAAGACGATCAGATACCGTCCTAGTTCTGACTATAAACGATGCCAACCAGCGATCGGGGGGCGTTAGTTTGACGACCTCCCCGGCAGGGGAAACCAAAGTCTTTGGGTTCCGGGGGAAGTATGGTTGCAAAGCTGAAACTTAAAGGAATTGACGGAAGGGCACCACCAGGCGTGGAGCCTGCGGCTTAATTTGACTCAACACGGGGAATCTCCGGCCCGGACACAGTGAGGATGACAGATTGAGAGCTCTTTCTTGATTCTGTG

>Ciona_savignyi

GCTTTTGTTATAATTTTTTTTTTTGTTATACCTATTATAATTAGAGGTTTTAGAAATTGATTAATTCCTATAATAATTGGAGCTCCGGATATAGCGTTTCCTCGAATAAATAATATAAGCTTTTGATTATTACCTCCTGCTTTTTTTATGTTATTAAAAAGTAGTTTTGCAGGAGCAAGAGTAAGAACTGGATGGACAGTTTATCCTCCTTTGTCTGCTACGGTAAGACATGCTCATTCTAGAGTAGATATAGCTATTTTTTCATTACATTTAGCTAGGGTTTCTAGTATTTTGAGATCTGTAAATTTTTTAGTTTTATTCAATATAAAAAATAAAAAATCGATAAGAGATTTAAGTTTGTTTTGTTGATCTTTG------------------------------------------------------------------------------------------------------------------------------------------------------------------------------------------------------------------------------------------------------------------------------------------------------------------------------------------------------------------------------------------------------------------------------------------------------------------------------------------------------------------------------------------------------------------------------------------------------------------------------------------------------------------------------------------------------------------------------------------------------------------------------------------------------------------------------------------------------------------------------------------------------------------------------------------------------------------------------------------------------------------

>Ciona_intestinalis

GCATTTGTAATAATTTTTTTTTTTGTTATACCAATTATAATTAGAAGATTTAGAAATTGATTAATTCCCCTAATAGTAAGAGCTCCTGATATGGCTTTCCCTCGGATAAATAATATAAGTTTTTGATTATTGCCTCCCTCTTTCTTTTTATTATTGAAAAGTAGATTCTCAGGGTCAAGAGTAAGAACAAGATGAACAGTTTATCCTCCTTTGTCCTCTAATATTGGACATTCTAATTCTAGGGTTGATATAGCTATTTTTTCTTTGCATTTAGCTGGGGTTTCTAGTATTTTAAGATCAGTTAATTTCTTAGTTTTATTTAATATAAAGAATAAAAAGTCTATAAGTAACTTAAGTTTATTTTGTTGATCTTTATTCTAGAGCTAATACATGCAAGGCGTGCTTTTATCAGACTGGTGACTCTGGATAACCTGGCGGATCGCACGGTCTGACGGATCATTCAAGTGTCTGCCCTATCAACTTTCGTCGGTACGGTATTCGCCTACCGAGTTCTTACGGGTAACGGGGAATCAGGGTTCGATTCCGGAGAGGGAGCCTGAGAAACGGCTACCACATCCAAGGAAGGCAGCAGGCGCGCAAATTACCCATTCCGACGCGGGGAGGTAGTGACGAAAAATAACAATTCGAGGCCCTGTAATATGAGTACACTTTAAAAGCTTTAACGAGTATCCATTGGAGGGCAAGTCTGGTGCCAGCAGCCGCGGTAATTCCAGCTCCAAAAGTATATATTTAAGTTGTTGCGGTTGAAAAGCTCGTAGTTGGATTTTGGGCGCGGGCGGTCGGTCCGTCGCGAGGCGTGACTGGTCGACCCGGGTCCGGTTCTCCGGCGGTGCTCTTGACTGAGTGTCGGCGGCGGCCGGAAAGTTTACTTTGAAAAAATTAGAGTGTTCAAAGCAGGCTCTCAGCCTGAATAATGGTGCATGGAATAATGGAATAGGACCTCGGTTCTATTTTGTTGGTTTTCGAGGTAATGATTAAGAGGGACGGGGGCATTCGTTTCTTGGATCGGCGAACGACTGCGAAAGCATTTGCCAAGAATGTTTAATCAAGAGCGAAAGTCAGAGGTTCGAAGACGATCAGATACCGTCCTAGTTCTGACTATAAACGATGCCAACTAGCGATCGGGGAGCGTTAATTTGACGACCTCCCCGGCAGGGGAAACCAAAGTCTTTGGGTTCCGGGGGAAGTATGGTTGCAAAGCTGAAACTTAAAGGAATTGACGGAAGGGCACCACCAGGAGTGGAGCCTGCGGCTTAATTTGACTCAACACGGGAAATCTCCGGCCCGGACACAGTGAGGATGACAGATTGAGAGCTCTTTCTTGATTCTGTG

>Botryllus_schlosseri

GCTTTTGTGATGATTTTCTTTTTTGTTATACCTATGATAGTTAGGAGGTTTGGTAATTGGTTATTACCTTTGATAGTGGGGAGTCCAGATATGGCTTTTCCTCGATTAAATAATATAAGTTTTTGATTGTTGCCCCCTGCTTTGTTTTTTCTTTTTAGAAGTTCTATAATTGAAAGTGGAGTTAGGACTGGGTGAACTGTTTATCCTCCCCTTTCTAGAAATCTAGCTCATTCTAGAGCTGCTTTGGATTGTGCTATTTTTTCTTTACATTTGGCTAGAGTGTCTAGTATTTTAAGATCTCTTAACTTTATGACTTTGTTTAATATAAAGGTAAAATGGGGACTCTTTTCTATATCTTTGTTTTGTTGAACTGTATTCCAGAGCTAATACATGCAAGGCGTGCTTTTATTGGTTTGATGACTCTGGATAACCACGCGGATCGCGCGGTCTGACAAACCATTCAAGTGTCTGACCTATCAACTTTCGAAGGTAAGCTACGGGCTTACCTTGTGATAACGGGTGACGGGGAATCAGGGTTCGATTCCGGAGAGGGAGCCTGAGAAACGGCTACCACATCCAAGGAAGGCAGCAGGCGCGCAAATTACCCATTCCGACACGGGGAGGTAGTGACGAAAAATAACAAAACGAGGCCCTGTAATATGAGTACATTCTAAAACTCTTAACGAGTATCCATTGGAGGGCAAGTCTGGTGCCAGCAGCCGCGGTAATTCCAGCTCCAAAAGTGTATGCTAAAGTTGTTGCGGTTGAAAAGCTCGTAGTTGGATATTGGGCGAGCGCGGTCGGTCCGTCGCAGGGCGTGACTGGTCGCGTTCGCTTCGGTTCTCCGTCGGTGCTCTTGACTGAGTGTCGGCGGTGGCCGAGAAGTTTACTTTGAAAAAATTAGAGTGTTCAAAGCAGGCTGGTCGCCTGAATAGTGTTGCATGGAATAATGGAATAGGACCTCGGTTCTATTTTGTTGGTTTTCGAGGTAATGATTAAGAGGGACGGGGGTGTCCGTTTCTTGGATCGGCGAACTACTGCGAAAGCATTCACCAAGAATGTTTAATCAAGAGCGAAAGTCAGAGGTTCGAAGACGATCAGATACCGTCCTAGTTCTGACTATAAACGATGCCAACTAGCGATCGGGAGGCGTTACCATGACGACCTTCCCGGCAGGGGAAACCAAAGTCTTTGGGTTCCGGGGGAAGTATGGTTGCAAAGCTGAAACTTAAAGGAATTGACGGAAGGGCACCACCAGGAGTGGAGCCTGCGGCTTAATTTGACTCAACACGGGGAAACTCCGGCCCGGACACAGTAAGGATGACAGATTGAGAGCTCTTTCATGATTCTGTG

>Botrylloides_leachii

GCTTTTGTAATAATTTTTTTCTTTGTTATACCTATGATGATTAGGGGGTTTGGTAACTGATTGTTACCTCTTATAGTGGGGAGTCCAGATATAGCTTTCCCACGTTTAAATAATATGAGTTTTTGGTTATTGCCTCCTGCACTATTTTTTCTTTTCATTAGTTCTATAATTGAAAGTGGGGTGGGAACGGGTTGGACAGTCTATCCCCCTTTGTCAAGAAATTTAGCTCATTCTGGGGCTGCATTAGATTGTGCTATTTTTTCACTTCATTTGGCTAGGGTTTCTAGTATTTTAAGGTCTTTAAATTTTATAACTTTGTTTAATATAAAAGTTAAGTGAGGGATGTTCTCTATATCTCTGTTTTGTTGAACTGTATTCCAGAGCTAATACATGCAAGGCGTGCTTTTATTGGTTTGATGACTCTGGATAACCACGCGGATCGCGCGGTCTGACAAACCATTCAAGTGTCTGACCTATCAACTTTCGAAGGTAAGCTACGGGCTTACCTTGTGATAACGGGTGACGGGGAATCAGGGTTCGATTCCGGAGAGGGAGCCTGAGAAACGGCTACCACATCCAAGGAAGGCAGCAGGCGCGCAAATTACCCATTCCGACACGGGGAGGTAGTGACGAAAAATAACAAAACGAGGCCCTGTAATATGAGTACATTCTAAAACTCTTAACGAGTATCCATTGGAGGGCAAGTCTGGTGCCAGCAGCCGCGGTAATTCCAGCTCCAACAGTGTATGCTAAAGTTGTTGCGGTTGAAAAGCTCGTAGTTGGATATTGGGCGAGCGCGGTCGGTCCGTCGCAGGGCGTGACTGGTCGCGTTCGCTTCGGTTCTCCGTCGGTGCTCTTGACTGAGTGTCGGCGGTGGCCGAGAAGTTTACTTTGAAAAAATTAGAGTGTTCAAAGCAGGCTGGTCGCCTGTATAGTGTTGCATGGAATAATGGAATAGGACCTCGGTTCTATTTTGTTGGTTTTCGAGGTAATGATTAAGAGGGACGGGGGTGTCCGTTTCTTGGATCGGCGAACTACTGCGAAAGCATTCACCAAGAATGTTTAATCAAGAGCGAAAGTCAGAGGTTCGAAGACGATCAGATACCGTCCTAGTTCTGACTATAAACGATGCCAACTAGCGATCGGGAGGCGTTACCATGACGACCTTCCCGGCAGGGGAAACCAAAGTCTTTGGGTTCCGGGGGAAGTATGGTTGCAAAGCTGAAACTTAAAGGAATTGACGGAAGGGCACCACCAGGAGTGGAGCCTGCGGCTTAATTTGACTCAACACGGGGAAACTCCGGCCCGGACACAGGAAGGATGACAGATTGAGAGCTCTTTCATGATTCTGTG

>Ascidiella_aspersa

GCTTTTGTTATGATTTTTTTTTTTGTTATGCCTATTATAATTGGCGGGTTTGGTAATTGGTTGTTGCCTATAATAGTTGGTGCTCCAGATATGGCTTTTCCTCGTATGAATAATATGAGTTTTTGACTACTTCCACCTGCATTTTTTATATTGATTATTTCTTCCATGATTAGGACTAGGGCTGGGACGGGATGGACAGTGTATCCCCCTCTCTCAGGTAACTTAGCTCATGCGGGCCCGGCTGTTGATTGTGCAATTTTTTCTTTACACCTGGCTGGTGTTTCTAGTATTCTCGGTTCTATTAATTTCTTAGTGATGATGGGTATGAAGGCGGGGTTTAGTTATTTAAACTTAAGTTTATTTTGCTGGTCTGTATGCTAGAGCTAATACATGCGAGGCGTGCTTTTATCAGTTTGGTGACTCTGGATAACTTTGCGGATCGCACGGTCTGACGGATCATTCAAGTGTCTGCCCTATCAACTTTCGTCGGTACGGTACTGGCCTACCGAGTTCTTACGGGTAACGGAGAATCAGGGTTCGATTCCGGAGAGGGAGCCTGAGAAACGGCTACCACATCCAAGGAAGGCAGCAGGCGCGCAAATTACCCATTCCGACGCGGGGAGGTAGTGACGAAAAATAACAAAACGAGGCCCTGTAATATGAGTACACTTTAAAACCTTTAACGAGTATCCATTGGAGGGCAAGTCTGGTGCCAGCAGCCGCGGTAATTCCAGCTCCAAAAGTGTATATTTAAGTTGTTGCGGTTAAAAAGCTCGTAGTTGGATTTTGGGCGCAGGCGGCCGGTCCGTCGCAAGGCGTGACTGGCCGTCCTGGCTCCGGTTCTCCGCCGGTGCCCTTGACTGAGTGTCGGCGGCGGCCGGAACGTTTACTTTGAAAAAATTAGAGTGTTCAAAGCAGGCGATTCGCCTGAATAATGGTGCATGGAATAATGGAATAGGACCTCGGTTCTATTTTGTTGGTTTTCGAGGTAATGATTAAGAGGGACGGGGGCATTCGTTTCTTGGATCGGCGAACGACTGCGAAAGCATTTGCCAAGAATGTTTAATCAAGAGCGAAAGTCAGAGGTTCGAAGACGATCAGATACCGTCCTAGTTCTGACTATAAACGATGCCAACTAGCGATCGGGGGGCGTTAGTTTTACGACCTCTCCGGCAGGGGAAACCAAAGTCTTTGGGTTCCGGGGGAAGTATGGTTGCAAAGCTGAAACTTAAAGGAATTGACGGAAGGGCACCACCAGGAGTGGAGCCTGCGGCTTAATTTGACTCAACACGGGAAATCTCCGGCCCGGACACAGTGAGGATGACAGATTGAGAGCTCTTTCTTGATTCTGTG

>Herdmania_momus

GCTTTTGTTATGATTTTTTTTTTTGTTATGCCAATTATAATTGGCAGATTTGGTAATTGATTGTTGCCTTTGATGGTGGGGAGTCCAGACATGGCATTTCCTCGGATGAATAATATGAGTTTTTGGTTATTACCTCCGGCTTTGTTTTTACTGTTAATTAGCTCTGTTATCGAGGCTGGGGTTGGTACAGGTTGAACTGTTTATCCCCCTTTGGCTGGGAATTTAGCACATTCAGGTCCAAGGTTAGATTGTGCAATTTTTTCATTACATTTAGCTAGGGTGTCTAGCATTTTAAGGTCTCTAAACTTCATGACGTTGTTTAACATAAAGAGTAAGTGGGGTATATTTTCAATACCTTTGTTTTGTTGGACTGTATTCTAGAGCTAATACATGCAAGGCGTGCTTTTATCAGTTTGATGACTCTGGATAACCATGCGGATCGCATGGTCTGACGAATCATTCAAGTGTCTGCCCTATCAACTTTCGAAGGTACGTTACGTGCCTACCTTGTGATAACGGGTGACGGGGAATCAGGGTTCGATTCCGGAGAGGGAGCCTGAGAGACGGCTACCACATCCAAGGAAGGCAGCAGGCGCGCAAATTACCCATTCCGACACGGGGAGGTAGTGACGAAAAATAACAAAACGAGGCTCTGTAATATGAGTACATTCTAAAACTCTTAACGAGTATCCATTGGAGGGCAAGTCTGGTGCCAGCAGCCGCGGTAATTCCAGCTCCAACAGTGTATGCTAAAGTTGTTGCGGTTGAAAAGCTCGTAGTTGGATCTTGGGTGGGTGTCGTCGGTCCGTCGCAAGGCGTGACTGGCGGCGCCTGATTCGGTTCTCCGTCGGTGCTCTTGACTGAGTGTCGGCGCTGGCCGGAAAGTTTACTTTGAAAAAATTAGAGTGTTCAAAGCAGGCTGTTCGCCTGCATAGTGTTGCATGGAATAATGGGATAGGACCTCGGTTCTATTTTGTTGGTTTTCGAGGTAATGATTAAGAGAGACGGGGGCGTCCGTTTCTTGGATCGGCGAACTACTGCGAAAGCATTCGCCAAGAATGTTTAATCAAGAGCGAAAGTCAGAGGTTCAAAGACGATCAGATACCGTCCTAGTTCTGACTATAAACGATGCCAACTAGCGATCGGGAGGCGTTACCATGACGACCTTCCCGGCAGGGGAAACCAAAGTCTTTGGGTTCTGGGGGAAGTATGGTTGCAAAGCTGAAACTTAAAGGAATTGACGGAAGGGCACCACCAGGAGTGGAGCCTGCGGCTTAATTTGACTCAACACGGGGAAACTCCGGCCCGGACACAGGAAGGATGACAGATTGAGAGCTCTTTCTTGATTCTGTG

>Phallusia_fumigata

GCCTTCGTTATAATTTTTTTCTTTGTGATACCCATTATGATTGGGAGGTTTGGTAATTGGCTCTTGCCTATAATAGTGGGGGCGCCTGACATGGCTTTTCCTCGTTTAAATAACATGAGTTTCTGATTGTTGCCTCCAGCATTGTTCTTGCTGTTGGTATCCTCTGTGGTGGGGAGTGGAGCTGGAACAAGGTGAACCGTCTACCCGCCGCTTGCTAGGAATCTAGCGCATTCCAGGGCCTCGGTTGACTGTGCTATTTTTTCACTCCACTTGGCAAGAGTTTCAAGTATTTTAGGTTCTATTAACTTTTTAGTTATGATAAATATAAAGGCTAAGTTTAGGACTTACAACATTAGTTTGTTTTGTTGGTCAGTTATCTAGAGCTAATACATGCGGGGCGTGCATTTATCATCTTGGCGACTCTGGGTAACTCAGCGGATCGCACGGTCTGACGGATCATTCAAGTGTCTGCCCTATCAACTGTCGTCGGTAGGGTATCGGCCTACCGTGTTCTTACGGGTGACGGGGAATCAGGGTTCGATTCCGGAGAGGGAGCCTGAGAAACGGCTACCACATCCAAGGAAGGCAGCAGGCGCGCAAATTACCCATTCCGACGCGGGGAGGTAGTGACGAAAAGTAACAAAAACAGGCCCTGTAATGCAATAGCCACTTTAAACCTTGAACGAGTATCCATTGGAGGGCAAGTCTGGTGCCAGCAGCCGCGGTAATTCCAGCTCCAAAAGTGTATATTTAAGTTGTTGCGGTTAAAAAGCTCGTAGTTGGATCTTGGGCTCAGGCGGCCGGTCCGTCGCAAGGCGTGACTGGCCGCACCTGCCCCGGCGCTCCGCCGGTGCCCTTAACTGAGTGTCGGCGGCGGCCGGGACGTTTACTTTGAAAAAATTAGAGTGTTCAAAGCAGGCGGCTCGCCTGGATAATGGTGCATGGAATAATGGAATAGGACCTCGGTTCTATTTTGTTGGTTTTCGAGGTAATGATTAAGAGGGACGGGGGCATTCGTTTCTTGGATCGGCGAGCGACTGCGAAAGCATTTGCCAAGAATGTTTAATCAAGAGCGAAAGTCAGAGGTTCGAAGACGATCAGATACCGTCCTAGTTCTGACTATAAACGATGCCAACCAGCGATCGGGGGGCGTTAGTTTGACGACCTCTCCGGCAGGGGAAACCAAAGTCTTTGGGTTCCGGGGGAAGTATGGTTGCAAAGCTGAAACTTAAAGGAATTGACGGAAGGGCACCACCAGGCGTGGAGCCTGCCGGCTTATTTGACTCAACACGGGGAATCTACCGGCCGGACACAGTGAGGATGACAGATTGAGAGCTCTTTCTTGATTCTGTG

>H1

GCTTTTGTTATAATTTTTTTTTTTGTTATACCTATTATAATTAGAGGTTTTAGAAATTGATTAATTCCTATAATAATTGGAGCTCCGGATATAGCGTTTCCTCGAATAAATAATATAAGCTTTTGATTATTACCTCCTGCTTTTTTTATGTTATTAAAAAGTAGTTTTGCAGGAGCAAGAGTAAGAACTGGATGGACAGTTTATCCTCCTTTGTCTGCTACGGTAAGACATGCTCATTCTAGAGTAGATATAGCTATTTTTTCATTACATTTAGCTAGGGTTTCTAGTATTTTGAGATCTGTAAATTTTTTAGTTTTATTCAATATAAAAAATAAAAAATCAATAAGAGATTTAAGTTTGTTTTGTTGATCTTTGTTCTAGAGCTAATACATGCGAGGCGTGCATTTATCAGCTTGGTGACTCTGGATAACTTTGCGGATCGCACGGTCTGACGGATCATTCAAGTGTCTGCCCTATCAACTTTCGTCGGTACGGTATTTGCCTACCGAGTTCTTACGGGTGACGGGGAATCAGGGTTCGATTCCGGAGAGGGAGCCTGAGAAACGGCTACCACATCCAAGGAAGGCAGCAGGCGCGCAAATTACCCATTCCGACGCGGGGAGGTAGTGACGAAAAATAACAATTCGAGGCCCTGTAATATGAGTACACTTTAAAAGCTTTAACGAGTATCCATTGGAGGGCAAGTCTGGTGCCAGCAGCCGCGGTAATTCCAGCTCCAAAAGTATATATTTAAGTTGTTGCGGTTAAAAAGCTCGTAGTTGGATTTTGGGCGCAGGCGGCCGGTCCGTCGCAAGGCGTGACTGGACGTCCTGGCTCCGGTTCTCCGCCGGTGCTCTTGACTGAGTGTCGGCGGCGGCCGGAACGTTTACTTTGAAAAAATTAGAGTGTTCAAAGCAGGCTCTCAGCCTGGATAATGGTGCATGGAATAATGGAATAGGACCTCGGTTCTATTTTGTTGGTTTTCGAGGTAATGATTAAGAGGGACGGGGGCATTCGTTTCTTGGATCGGCGAACGACTGCGAAAGCATTTGCCAAGAATGTTTAATCAAGAGCGAAAGTCAGAGGTTCGAAGACGATCAGATACCGTCCTAGTTCTGACTATAAACGATGCCAACTAGCGATCGGGGGGCGTTACATTGACGACCCCTCCGGCAGGGGAAACCAAAGTCTTTGGGTTCCGGGGGAAGTATGGTTGCAAAGCTGAAACTTAAAGGAATTGACGGAAGGGCACCACCAGGAGTGGAGCCTGCGGCTTAATTTGACTCAACACGGGAAATCTCCGGCCCGGACACAGTGAGGATGACAGATTGAGAGCTCTTTCTTGATTCTGTG

>H12

GCGTTTGTTATAATTTTTTTTTTTGTTATACCCACAATGATTGGGGGTTTTAGGAATTGGCTCTTGCCTATGATAGTTAGAGCTCCGGATATAGCCTTTCCTCGATTAAATAATATGAGCTTCTGGCTTTTGCCTCCAGCATTTTTTTTTATAGTTATATCTACCTTAATGGGTAGGGGTGCAAGAACGGGATGAACTGTTTACCCTCCCCTTTCAAGAGGTGTGGCCCACAGCGGTCCTGCAGTGGATTTTGCTATTTTTTCCCTCCATCTTGCTGGTATTTCGAGCATTCTAGGATCTATTAATTTTCTTGTTATGTGGAATATGAAGGCTAAGATAGAGGCCTATCATATTAGTTTGTTCTGTTGGTCAGTTATCTAGAGCTAATACATGCGGGGCGTGCATTTATCAGTTTGGTGACTCTGGGTAACTCAGCGGATCGCACGGTCTGACGGATCATTCAAGTGTCTGCCCTATCAACTTTCGTCGGTAGGGTATCGGCCTACCGAGTTGTTACGGGTGACGGGGAATCAGGGTTCGATTCCGGAGAGGGAGCCTGAGAAACGGCTACCACATCCAAGGAAGGCAGCAGGCGCGCAAATTACCCATTCCGACGCGGGGAGGTAGTGACGAAAAATAACAAAACGAGGCCCTGTAATATGAGCACACTTTAAAACCTTTAACGAGTATCCATTGGAGGGCAAGTCTGGTGCCAGCAGCCGCGGTAATTCCAGCTCCAAAAGTGTATATTTAAGTTGTTGCGGTTAAAAAGCTCGTAGTTGGATCTTGGGCGCAGGCGGCCGGTCCGTCGCAAGGCGTGACTGGCCGCACCTGCCCCGGCGCTCCGCCGGTGCCCTTGACTGAGTGTCGGCGGCGGCCGGGACGTTTACTTTGAAAAAATTAGAGTGTTCAAAGCAGGCGACTCGCCTGAATAATGGTGCATGGAATAATGGAATAGGACCTCGGTTCTATTTTGTTGGTTTTCGAGGTAATGATTAAGAGGGACGGGGGCATTCGTTTCTTGGATCGGCGAGCGACTGCGAAAGCATTTGCCAAGAATGTTTAATCAAGAGCGAAAGTCAGAGGTTCGAAGACGATCAGATACCGTCCTAGTTCTGACTATAAACGATGCCAACCAGCGATCGGGGGGCGTTAGTTTGACGACCTCCCCGGCAGGGGAAACCAAAGTCTTTGGGTTCCGGGGGAAGTATGGTTGCAAAGCTGAAACTTAAAGGAATTGACGGAAGGGCACCACCAGGCGTGGAGCCTGCGGCTTAATTTGACTCAACACGGGGAATCTCCGGCCCGGACACAGTGAGGATGACAGATTGAGAGCTCTTTCTTGATTCTGTG

>H17

GCGTTTGTTATAATTTTTTTTTTTGTTATACCCACAATGATTGGGGGTTTTAGGAATTGGCTCTTGCCTATGATAGTTAGAGCTCCGGATATAGCCTTTCCTCGATTAAATAATATGAGCTTCTGGCTTTTGCCTCCAGCATTTTTTTTTATAGTTATATCTACCTTAATAGGTAGGGGTGCAAGAACGGGATGAACTGTTTATCCTCCCCTTTCAAGAGGTGTGGCCCACAGCGGTCCTGCAGTGGATTTTGCTATTTTTTCCCTCCATCTTGCTGGTATTTCGAGTATTCTAGGATCTATTAATTTTCTTGTTATGTGGAATATGAAGGCTAAGATAGAGGCCTATCATATTAGTTTGTTTTGTTGGTCAGTTATCTAGAGCTAATACATGCGGGGCGTGCATTTATCAGTTTGGTGACTCTGGGTAACTCAGCGGATCGCACGGTCTGACGGATCATTCAAGTGTCTGCCCTATCAACTTTCGTCGGTAGGGTATCGGCCTACCGAGTTGTTACGGGTGACGGGGAATCAGGGTTCGATTCCGGAGAGGGAGCCTGAGAAACGGCTACCACATCCAAGGAAGGCAGCAGGCGCGCAAATTACCCATTCCGACGCGGGGAGGTAGTGACGAAAAATAACAAAACGAGGCCCTGTAATATGAGCACACTTTAAAACCTTTAACGAGTATCCATTGGAGGGCAAGTCTGGTGCCAGCAGCCGCGGTAATTCCAGCTCCAAAAGTGTATATTTAAGTTGTTGCGGTTAAAAAGCTCGTAGTTGGATCTTGGGCGCAGGCGGCCGGTCCGTCGCAAGGCGTGACTGGCCGCACCTGCCCCGGCGCTCCGCCGGTGCCCTTGACTGAGTGTCGGCGGCGGCCGGGACGTTTACTTTGAAAAAATTAGAGTGTTCAAAGCAGGCGACTCGCCTGAATAATGGTGCATGGAATAATGGAATAGGACCTCGGTTCTATTTTGTTGGTTTTCGAGGTAATGATTAAGAGGGACGGGGGCATTCGTTTCTTGGATCGGCGAGCGACTGCGAAAGCATTTGCCAAGAATGTTTAATCAAGAGCGAAAGTCAGAGGTTCGAAGACGATCAGATACCGTCCTAGTTCTGACTATAAACGATGCCAACCAGCGATCGGGGGGCGTTAGTTTGACGACCTCCCCGGCAGGGGAAACCAAAGTCTTTGGGTTCCGGGGGAAGTATGGTTGCAAAGCTGAAACTTAAAGGAATTGACGGAAGGGCACCACCAGGCGTGGAGCCTGCGGCTTAATTTGACTCAACACGGGGAATCTCCGGCCCGGACACAGTGAGGATGACAGATTGAGAGCTCTTTCTTGATTCTGTG

>S20

GCTTTTGTTATAATTTTTTTTTTTGTTATACCTATTATAATTAGAGGTTTTAGAAATTGATTAATTCCTATAATAATTGGAGCTCCGGATATAGCGTTTCCTCGAATAAATAATATAAGCTTTTGATTATTACCTCCTGCTTTTTTTATGTTATTAAAAAGTAGTTTTGCAGGAGCAAGAGTAAGAACTGGATGGACAGTTTATCCTCCTTTGTCTGCTACGGtAAGACATGCTCATTCTAGAGTAGATATAGCTATTTTTTCATTACaTTTAGCTAGGGTTTCTAGTATTTTGAGATCTGTAAATTTTTTAGTTTTATTCAATATAAAAAATAAAAAATCGATAAgAGATTTAAGTTTGTTTTGTTGATCTTTGTTCTAGAGcTAATACATGCGAGGCGTGCATTTATCAGCTTGGTGACTCTGGATAACTTTGCGGATCGCACGGTCTGACGGATCATTCAAGTGTCTGCCCTATCAACTTTTTCGGGTACGGTATTTGCCTACCGAGTTCTTACGGGTGACGGGGAATCAGGGTTCGATTCCGGAGAGGGAGCCTGAGAAACGGCTACCACATCCAAGGAAGGCAGCAGGCGCGCAAATTACCCATTCCGACGCGGGGAGGTAGTGACGAAAAATAACAATTCGAGGCCCTGTAATATGAGTACACTTTAAAAGCTTTAACGAGTATCCATTGGAGGGCAAGTCTGGTGCCAGCAGCCGCGGTAATTCCAGCTCCAAAAGTATATATTTAAGTTGTTGCGGTTAAAAAGCTCGTAGTTGGATTTTGGGCGCAGGCGGCCGGTCCGTCGCAAGGCGTGACTGGACGTCCTGGCTCCGGTTCTCCGCCGGTGCTCTTGACTGAGTGTCGGCGGCGGCCGGAACGTTTACTTTGAAAAAATTAGAGTGTTCAAAGCAGGCTCTCAGCCTGGATAATGGTGCATGGAATAATGGAATAGGACCTCGGTTCTATTTTGTTGGTTTTCGAGGTAATGATTAAgAGGGACGGGGGCATTCgTTTcTTGGATCGGCGAACGACTGCGAAAGCATTTGCCAAgAAtGTTTAATCAAgAGCGAAAGTCAgAGGTTCgAAGAaGATCAgATACCGTCCTAGTTCTGACTATAAACGATGCCAACTAGCGATCGGGGGGCGTTACaTTGACgACCCCTCcGGcaGGGGAAACCAAAGTCTTTGGGTTCCGGGGGAAGTATGGTTGCAAAGCTGAAACTTAAAGGAATTGACGGAaGGGCACCACCAGGAGTGGAGCCTGCGGCTTAaTTTGACTCAACACGGGAAATCTCCGGCCCGGACACAGTGAGGATGACAGATTGAGAGCTCTTTCTTGATTCTGTG

>S31

GCtTTGgTGAtGATTTTTTTTTTTGTTaTACCAgTCaTAGtGGGGGGCTTTGgAAATTGATTACTCCCTcTAATAATTGGGAGTCCGGATaTAGCATTTCCTCGTTTGAATAACATAAGTTTTTGGTTGTTACCACCCTCATTGTTTTTGCTAcTGGTAAGTTCAATAGTTGAGAGTGGTGTGGGGACAGGTTGAACTGTGTATCCTCCACTAGCTGGTaATCTGGcTCATTCAGGGCCAGCTTTGGATTGTGCTATTTTTTCTTTACACTTAGCTAGGGTGTCTAGTATTTTAGGATCTTTGAATTTTATAACTTTGTTTAATATGAAAAGAAAGTGGGATTTATTTATGATACCTTTaTTTTGTTGAACTATTTTCTAGAGCTAATACATGCGAGGCGTGCTTTTATCAGTTTGACGACTCTGGATAACCACGCGGATCGCATGGTCTGACGAATCATTCAAGTGTCTGCCCTATCAACTTTCGAAGGTACGTTACGTGCCTACCTTGTGATAACGGGTGACGGGGAATCAGGGTTCGATTCCGGAGAGGGAGCCTGAGAAACGGCTACCACATCCAAGGAAGGCAGCAGGCGCGCAAATTACCCATTCCGACACGGGGAGGTAGTGACGAAAAATAACAAAACGAGGCCCTGTAATATGAGTACATTCTAAACCTCTTAACGAGTATCCATTGGAGGGCAAGTCTGGTGCCAGCAGCCGCGGTAATTCCAGCTCCAACAGTGTATGCTAAAGTTGTTGCGGTTGAAAAGCTCGTAGTTGGATCTTGGGCGAGTGCCGCCGGTCCGTCGCAAGGCGTGACTGGCGGCGCTTGCGTCGGTTCTCCGTCGGTGCTCTTGACTGAGTGTCGGCGGTGGCCGAGAAGTTTACTTTGAAAAAATTAGAGTGTTCAAAGCAGGCTGGTCGCCTGCATAGTGTTGCATGGAATAATGGAATAGGACCTCGGTTCTATTTTGTTGGTTTTCGAGGTAATGATTAAGAGGGACGGGGGCGTCCGTTTCTTGGATCGGCGAACTACTGCGAAAGCATTCGCCAAGAATGTTTAATCAAGAGCGAAAGTCAGAGGTTCGAAGACGATCAGATACCGTCCTAGTTCTGACTATAAACGATGCCaACTAGCGATCGGGAGGCGTTACCATGACGACCTTCCCGGCAGGGGAAACCAAAGTCTTTGGGTTCCGGGGGAAGTATGGTTGCAAAGCTGAAACTTAAAGGAATTGACGGAAGGGCACCACCAGGAGTGGAGCCTGCGGCTTAATTTGACTCAaCACGGGGAAACTCCGGCCCGGACACAGGAAGGATGACAGATTGAGAGCTCTTTCTTGATTCTGTG

>S32

GCTTTCGTTATAATTTTTTTTTTTGTGATGCCTATTATAATTAGGGGATTTAGGAATTGGTTACTACCTTTAATAATTAGGAGTCCCGATATAGCATTTCCTCGATTGAATAACATAAGTTTTTGATTGTTACCTCCAGCTTTGATTTTATTATTAATAAGTTCTGTTGTTGATAATGGGGTTGGTACTGGTTGGACGGTTTATCCTCCTTTATCAGGAAATCTAGCACACTCTAGAGTATCTGTTGACTGTGCTATTTTTTCGTTACATTTAGCAGGTATGTCTAGTATTTTAGGATCTTTAAATTTTATAACTTTGTTTAATATAAAGAGATCTTGGAGTATGTTTTCAATGACTCTATTTTGTTGAACTGTATTCTAGAGCTAATACATGCGAGGCGTGCTTTTATCAGTTTGACGAGTCTGGATAACCACGCGGATCGTACGGTCTGACCCATCATTCAAGTGTCTGACCTATCAACTTTCGAAGGTACGCTACGTGCCTACCTTGTGATAACGGGTAACGGGGAATCAGGGTTCGATTCCGGAGAGGGAGCCTGAGAAACGGCTACCACATCCAAGGAAGGCAGCAGGCGCGCAAATTACCCATTCCGACACGGGGAGGTAGTGACGAAAAATAACAAAACGAGGCCCTGTAATATGAGTACATTCTAAAACTCTTAACGAGTATCCATTGGAGGGCAAGTCTGGTGCCAGCAGCCGCGGTAATTCCAGCTCCAACAGTGTATGCTAAAGTTGTTGCGGTTGAAAAGCTCGTAGTTGGATTTTGGGCGAGCGCGGCTGGTCCGTCGCAGGGCGTGACTGGCCGCGTTCGCTTCGGTTCTCTGTCGGTGCTCTTGACTGAGTGTCGGCGGTGACCGATAAGTTTACTTTGAAAAAATTAGAGTGTTCAAAGCAGGCTGCTCGCCTGAATAGTGTTGCATGGAATAATGGAATAGGACCTCGGTTCTATTTTGTTGGTTTTCGAGGTAATGATTAAGAGGGACGGGGGCGTCCGTTTCTTGGATCGGCGAACTACTGCGAAAGCATTCGCCAAGAATGTTTAATCAAGAGCGAAAGTCAGAGGTTCGAAGACGATCAGATACCGTCCTAGTTCTGACTATAAACGATGCCAACTAGCGATCGGGAGGCGTTACCATGACGACCTTCCCGGCAGGGGAAACCAAAGTCTTTGGGTTCTGGGGGAAGTATGGTTGCAAAGCTGAAACTTAAAGGAATTGACGGAAGGGCACCACCAGGAGTGGAGCCTGCGGCTTAATTTGACTCAACACGGGGAAACTCCGGCCCGGACACAGGTAGGATGACAGATTGAGAGCTCTTTCTTGATTCTGTG

>S37

GCTTtTGTTATAATTTTTTTTTTTGTTATACCTaTTATAATTAGAGGtTTTAGAAATTGATTAATTCCTATaATAATTGGAGcTCCGGATaTAGCGTTtCCTCGAATAAATAAtATAAGCTTTTGATTATTACCTCCTGCTTTTTTTATGTTaTTAAAAAGTAGTTTTGCAGGAgCAAGAGTAAGAACTGGATGGACAGTTTATCCTCCTTTGTCTGCTACGGTAAGAcATGCTCATTCTAgAGTAGAtAtAGCTATTTTTTCATTACATTtAGCTAGGGTTTCTAGTATTTTGAgAtCTGTAAATTTTTTAGTTTTATTCAATATAAAAAATAAAAAATCGATAAGAGATTTAAGTTTGTTTTGTTGATCTTTGTTCTAGAGCTAATACATGCGAGGCGTGCATTTATCAGCTTGGTGACTCTGGATAACTTTGCGGATCGCACGGTCTGACGGATCATTCAAGTGTCTGCCCTATCAACTTTCGTCGGTACGGTATTTGCCTACCGAGTTCTTACGGGTGACGGGGAATCAGGGTTCGATTCCGGAGAGGGAGCCTGAGAAACGGCTACCACATCCAAGGAAGGCAGCAGGCGCGCAAATTACCCATTCCGACGCGGGGAGGTAGTGACGAAAAATAACAATTCGAGGCCCTGTAATATGAGTACACTTTAAAAGCTTTAACGAGTATCCATTGGAGGGCAAGTCTGGTGCCAGCAGCCGCGGTAATTCCAGCTCCAAAAGTATATATTTAAGTTGTTGCGGTTAAAAAGCTCGTAGTTGGATTTTGGGCGCAGGCGGCCGGTCCGTCGCAAGGCGTGACTGGACGTCCTGGCTCCGGTTCTCCGCCGGTGCTCTTGACTGAGTGTCGGCGGCGGCCGGAACGTTTACTTTGAAAAAATTAGAGTGTTCAAAGCAGGCTCTCAGCCTGGATAATGGTGCATGGAATAATGGAATAGGACCTCGGTTCTATTTTGTTGGTTTTCGAGGTAATGATTAAGAGGGACGGGGGCATTCGTTTCTTGGATCGGCGAACGACTGCGAAAGCATTTGCCAAGAATGTTTAATCAAGAGCGAAAGTCAGAGGTTCGAAGACGATCAGATACCGTCCTAGTTCTGACTATAAACGATGCCAACTAGCGATCGGGGGGCGTTACATTGACGACCCCTCCGGCAGGGGAAACCAAAGTCTTTGGGTTCCGGGGGAAGTATGGTTGCAAAGCTGAAACTTAAAGGAATTGACGGAAGGGCACCACCAGGAGTGGAGCCTGCGGCTTAATTTGACTCAACACGGGAAATCTCCGGCCCGGACACAGTGAGGATGACAGATTGAGAGCTCTTTCTTGATTCTGTG

>S43

GCTTTGgtGATGATTTTTTTTTTTGTTATACCAGtCaTAgTGGGGGGCtTTGGAAATTgATTaCTCCCAcTAaTAAtTGGGAGtCCAgATATAGCATTTCCTCGTTTGAATAACATAAGTTTTTGGTTGTTACCACCCtCATTGtTTTTGcTGCTGGtAAGTTcAATAgtTGAGAGTGGTGTGGGGACAGGTTGAACTGTGTATCCTCCACTAcCTGGTAATCTGgCTCATTCAGGGCCAGCTTTGGATTGTGCTATTTTTTCTTTACACTTAGCTAGGGtGTCTAGTATTTtAGGATCTTTAAATTTTATAAcTTTGTTTAATATgAAAAgAAAGTGGGATTTAttTAtGATACCTTTatTTTGTTGAACTATTTTCTAGAGCTAATACATGCGAGGCGTGCTTTTATCAGTTTGACGACTCTGGATAACCACGCGGATCGCATGGTCTGACGAATCATTCAAGTGTCTGCCCTATCAACTTTCGAAGGTACGTTACGTGCCTACCTTGTGATAACGGGTGACGGGGAATCAGGGTTCGATTCCGGAGAGGGAGCCTGAGAAACGGCTACCACATCCAAGGAAGGCAGCAGGCGCGCAAATTACCCATTCCGACACGGGGAGGTAGTGACGAAAAATAACAAAACGAGGCCCTGTAATATGAGTACATTCTAAACCTCTTAACGAGTATCCATTGGAGGGCAAGTCTGGTGCCAGCAGCCGCGGTAATTCCAGCTCCAACAGTGTATGCTAAAGTTGTTGCGGTTGAAAAGCTCGTAGTTGGATCTTGGGCGAGTGCCGCCGGTCCGTCGCAAGGCGTGACTGGCGGCGCTTGCGTCGGTTCTCCGTCGGTGCTCTTGACTGAGTGTCGGCGGTGGCCGAGAAGTTTACTTTGAAAAAATTAGAGTGTTCAAAGCAGGCTGGTCGCCTGCATAGTGTTGCATGGAATAATGGAATAGGACCTCGGTTCTATTTTGTTGGTTTTCGAGGTAATGATTAAGAGGGACGGGGGCGTCCGTTTCTTGGATCGGCGAACTACTGCGAAAGCATTCGCCAAGAATGTTTAATCAAGAGCGAAAGTCAGAGGTTCGAAGACGATCAGATACCGTCCTAGTTCTGACTATAAACGATGCCAACTAGCGATCGGGAGGCGTTACCATGACGACCTTCCCGGCAGGGGAAACCAAAGTCTTTGGGTTCCGGGGGAAGTATGGTTGCAAAGCTGAAACTTAAAGGAATTGACGGAAGGGCACCACCAGGAGTGGAGCCTGCGGCTTAATTTGACTCAACACGGGGAAACTCCGGCCCGGACACAGGAAGGATGACAGATTGAGAGCTCTTTCTTGATTCTGTG

>S44

GCtTTGGTGATGaTTTTTTTTTTTGTTaTACCAgtCaTAgtGGGGGGCTTgGGAAATtgATTaCTCCcACTaATAaTTGGGAGtCCAGATATAGcATTTCCTCGTTTGAATAAcATAAGTTTTTGGTTGTTACCACCtTCATTgTTTTTgTTACTGGTAAGTtcAATAgTTGAgAGTGGtGTGGGGACAGGTTGAACTGtGTATCCTCCACTAcCTGGTAATCtGGCTCATTCAgGGCCAGCTtTGGATTGaGCTATTTTTTCTTTacacAtcGCTaGGGtGtcTAGTATTTtAgGATCTTTAAATTTTATAACTTTGTTTAATaTGAAAAGAAAGTGGGATTTAaTTATGATACCGTTTATTTTGTTGGACTATTTCTAGAGCTAATACATGCGAGGCGTGCTTTTATCAGTTTGACGACTCTGGATAACCACGCGGATCGCATGGTCTGACGAATCATTCAAGTGTCTGCCCTATCAACTTTCGAAGGTACGTTACGTGCCTACCTTGTGATAACGGGTGACGGGGAATCAGGGTTCGATTCCGGAGAGGGAGCCTGAGAAACGGCTACCACATCCAAGGAAGGCAGCAGGCGCGCAAATTACCCATTCCGACACGGGGAGGTAGTGACGAAAAATAACAAAACGAGGCCCTGTAATATGAGTACATTCTAAACCTCTTAACGAGTATCCATTGGAGGGCAAGTCTGGTGCCAGCAGCCGCGGTAATTCCAGCTCCAACAGTGTATGCTAAAGTTGTTGCGGTTGAAAAGCTCGTAGTTGGATCTTGGGCGAGTGCCGCCGGTCCGTCGCAAGGCGTGACTGGCGGCGCTTGCGTCGGTTCTCCGTCGGTGCTCTTGACTGAGTGTCGGCGGTGGCCGAGAAGTTTACTTTGAAAAAATTAGAGTGTTCAAAGCAGGCTGGTCGCCTGCATAGTGTTGCATGGAATAATGGAATAGGACCTCGGTTCTATTTTGTTGGTTTTCGAGGTAATGATTAAGAGGGACGGGGGCGTCCGTTTCTTGGATCGGCGAACTACTGCGAAAGCATTCGCCAAGAATGTTTAATCAAGAGCGAAAGTCAGAGGTTCGAAGACGATCAGATACCGTCCTAGTTCTGACTATAAACGATGCCAACTAGCGATCGGGAGGCGTTACCATGACGACCTTCCCGGCAGGGGAAACCAAAGTCTTTGGGTTCCGGGGGAAGTATGGTTGCAAAGCTGAAACTTAAAGGAATTGACGGAAGGGCACCACCAGGAGTGGAGCCTGCGGCTTAATTTGACTCAACACGGGGAAACTCCGGCCCGGACACAGGAAGGATGACAGATTGAGAGCTCTTTCTTGATTCTGTG

>Molgula

GCTTTAGTTATAATTTTTTTTTTTGTAATACCTATTACAATAAGGAGATTTGGGAATTGGCTAATTCCTCTTTTTATGAGATGTCCTGATATGGCTTTTCCTCGTATAAATAATTTTTCTTTCTGGTTACTTCCTTTTTCTTTTAGTTTATTATTACTTAGTGGTTTTATGAATATGAGAGTTGGGGCAGGGTGGACCATTTACCCTCCTCTATCTTCTATTTTGAGACATCCTAGAATTCAGATGGATTTTGCTATTTTTAGTCTACATTTGGCTAGAATTAGTAGTATTCTTTCTTCTATTAATTTTATAGTAATTTTAAATATATCTCCTAAAATAAAAATTTTTCATTTATCTTTAATAATGTGAAGTATTTTCTAGAGCTAATACATGCAAGGCGCGCACTTATCAGTTTGGTGACTCTGGATAATTCCGCTGATCGTATGGTCTGACGGATCATTCAAGTGTCTGCCCTATCAACTTTCGAATGTATGGTATTTGCCTACGTTGTGATAACGGGTGACGGGGAATCAGGGTTCGATTCCGGAGAGGGAGCCTGAGAAACGGCTACCACATCCAAGGAAGGCAGCAGGCGCGCAAATTACCCACTTTGAAATGAAGAGGTAGTGACGAAAAATAACAAAACGAGGCCCTGTAATATGAGTACAATTTAAACTCTTTAACGAGTATCCATTGGAGGGCAAGTCTGGTGCCAGCAGCCGCGGTAATTCCAGCTCCAATAGTGTATGCTAAAGTTGTTGCGGTTGAAAAGCTCGTAGTTGGATCTAGGGTGCTGGTCGGTGGTCCGCCGCAAGGTGTGACTGTCGGCCGGTCTTCTGGTTTTCCGGCGGTTCTCTTAACTGAGTGTCGTCGGTGGCCAGTGCGTTTACTTTGAAAAAATTAGAGTGTTTAAAGCAGGCTGTTTGCCTGAATATCGGTGCATGGAATAATGGAATAGGACCTCGAGTCTATTTTGTTGGTTTTCGAGGTAATGATTAAGAGGGACGGGGGCATCCGTTTCTTGGATCGGCGAACTATTGCGAAAGCATTTGCCAAGAATGTTTAATCAAGAGCGAAAGTCAGAGGTTCGAAGACGATCAGATACCGTCCTAGTTCTGACCATAAACGATGCCAACTAGCGATCCGCCGGCGTTACCATGACGACTCGGCGGGTAGGGGAAACCAAAGTCTTTGGGCTCCGGGGGAAGTATGGTTGCAAAGCTGAAACTTAAAGGAATTGACGGAAGGGCACCACCAGGAGTGGAGCCTGCGGCTTAATTTGACTCAACACGGGGAAACTCCGGCCCGGACACAGGTAGGATGACAGATTGAGAGCTCTTTCTTGATTCTGTG

>Branchiostoma

GCGTTTGTTATAATCTTCTTCATAGTTATGCCAATTATGATTGGGGGATTTGGGAATTGGTTAGTGCCCATGATAATTGGGGCGCCAGATATAGCTTTCCCCCGTATAAATAATATGAGCTTTTGAATGCTACCGCCGTCATTTTCTTTATTATTGGCGTCTTCTGCTGTCGAGGCTGGGGTTGGAACGGGTTGGACAGTTTACCCTCCTTTATCTAGTAACATTGCTCACGCAGGGGCATCTGTTGATCTGGCTATTTTCTCTCTACATTTGGCTGGTGTATCATCCATTTTAGGGGCAATCAATTTTATTACAATTCATAATATGCGTGCTAGCATTGAGTGAAATCGGGTGCCGTTGTTTGTTTGGTCAATTTTCTAGAGCTAATACATGCGTGGCGTGCATTTATCAGTTTGGTGACTCTGGATAACCCAGCCGATCGCACGGTCTGACAGATCATTCGAATGTCTGCCCTATCAACTTTCGATGGTAGGTTCTGTGCCTACCATGTGGCAACGGGTGACGGGGAATCAGGGTTCGGTTCCGGAGAGGGAGCCTGAGAAACGGCTACCACATCCAAGGAAGGCAGCAGGCGCGCAAATTACCCACTCCGACTCGGGGAGGTAGTGACGAAAAATAACAATTCGAGGCCCCGTAATATGGGTACACTTTAAATCCTTTAACGAGGATCTATTGGAGGGCAAGTCTGGTGCCAGCAGCCGCGGTAATTCCAGCTCCAATAGCGTATATTAAAGTTGTTGCGGTTAAAAAGCTCGTAGTTGGATCTTGGGACCGGGCTTGCGGTCCGCCGCGAGGCGTGACTGCTCGTCCCGGCGCCGGATTCTCCTTGGTGCTCTTAACTGAGTGCCTCGGGGTGCCGGAGCGTTTACTTTGAAAAAATTAGAGTGTTCAAAGCAGGCCTGGCGCCTGAATAGTGGTGCATGGAATAATGGAATAGGACCTCGGTTCTATTTCGTTGGTTTTCGAGGTAATGATCAAGAGGGACGGGGGCATTCGTTTCTTGGATCGCCGAACCACTGCGAAAGCATTTGCCAAGAATGATTGATCAAGAACGAAAGTTGTGGGCGCGAAGGCGATCAGATACCGCCCTAGTCACAACCATAAACGATGCCAACCAGCGATCCGCCGGCGTTACTTCGATGACCCGATGGGCAGGGGAAACCTGAGTTTTCGGGTTCCGGGGGAAGTATGGTTGCAAAGCTGGAACTTAAAGGAATTGACGGAAGGGCACCACCAGGAGTGGAGCCTGCGGCTTAATTTGACTCAACACGGGGAAACTCCGGCCCGGACACAGTAAGGATGACAGATTGAGAGCTCTTTCTTGATTCTGTG
