# Supplementary material for: Phylogenetic comparison of egg transparency in ascidians by hyperspectral imaging
Source: Sci Rep. 2020 Nov 30;10:20829. doi: 10.1038/s41598-020-77585-y (PMC7709464; doi:10.1038/s41598-020-77585-y)
Supplement: Supplementary file 2 — Supplementary Information 2. [file 41598_2020_77585_MOESM2_ESM.pdf]

Supplementary Information

Phylogenetic Comparison of Egg Transparency in Ascidians by Hyperspectral Imaging

Takumi T. Shito[1], Naohiro Hasegawa[2], Kotaro Oka[1,3,4]* and Kohji Hotta[1]*

1. Department of Bioscience and Informatics, Faculty of Science and Technology, Keio University, Yokohama, 223-8522, Japan
2. Department of Natural History Sciences, Graduate School of Science, Hokkaido University, Kita 10 Nishi 8 Kitaku, Sapporo, Hokkaido, 060-0810, Japan
3. Waseda Research Institute for Science and Engineering, Waseda University, 2-2 Wakamatsucho, Shinjuku, Tokyo, 162-8480, Japan
4. Graduate Institute of Medicine, College of Medicine, Kaohsiung Medical University, Kaohsiung City, 80708, Taiwan

*Corresponding Authors: khotta@bio.keio.ac.jp (K. Hotta) and oka@bio.keio.ac.jp (K. Oka)

Additional data 1:
File name: AdditionalFile1.docx
Sequence alignment of COI and 18S of different ascidian individuals.

Suppl. Table 1 Identification of sampled ascidians in different sampling sites.

order	family	genus	species *	individual ID and sampling sites		
				(1)	(2)	(3)
Phlebobranchia	Ascidiidae	*Ascidiella*	*aspersa*	s17: Sado	Z: Onagawa	
		Ascidia	*zara*	h12: Honmoku	h17: Honmoku	
	Cionidae	*Ciona*	*savignyi*	h1: Honmoku	s20: Sado	s37: Sado
			robusta	C: Aquacultured in Kyoto		
Stolidobranchia	Pyuridae	*Microcosmus*	*squamiger*	m3: Misaki		
		Halocynthia	*hispada*	s9: Sado		
		Pyura	sp.	s5: Sado		
		Herdmania	sp.	m4: Misaki		
	Styelidae	*Styela*	*plicata*	m1: Misaki		
		Polycarpa	*cryptocarpa*	m5: Misaki		
	Unidentified	Unidentified	sp. 1	s1: Sado	s4: Sado	s12: Sado
		Unidentified	sp. 2	s31: Sado		
		Unidentified	sp. 3	s32: Sado		
		Unidentified	sp. 4	s43: Sado	s44: Sado	

* specific epithet

Suppl. Table 1 Identification of sampled ascidians in different sampling sites.
The taxonomy and sampling sites of ascidians we collected. The taxonomy was determined based on anatomy and molecular information of COI, 18S sequence in Suppl. Table 3.

Suppl.Table 2 The best hit genes to the cloned genes in different ascidians.

	COI			18S		
No.	Species	per.ident	Accession	Species	per.ident	Accession
h1	*Ciona savignyi*	100%	JF919701.1	Uncultured eukaryote	99.75%	AB191429.1
h12	*Ascidia zara*	99.59%	AB794956.1	*Phallusia mammillata*	98.93%	AF236803.2
h17	*Ascidia zara*	100%	KY235396.1	*Phallusia mammillata*	98.88%	AF236803.2
m1	*Styela plicata*	100%	JX885716.1	*Styela plicata*	99.60%	KJ818250.1
m3	*Microcosmus squamiger*	100%	KF369151.1	*Microcosmus squamiger*	99.43%	FM244855.1
m4	*Herdmania momus*	85.83%	HM490296.1	*Herdmania* sp.	99.05%	FM244852.1
m5	*Cyanallagma interruptum* voucher	84.36%	MN346185.1	*Polycarpa mytiligera*	98.98%	FM244860.1
s1	*Herdmania grandis*	82.21%	FJ528630.1	N/A	N/A	N/A
s4	*Hydrobaenus laticaudus*	85.63%	MN082709.1	*Pyura dura*	97.28%	FM244856.1
s5	*Pyura mirabilis*	92.39%	LC432332.1	*Pyura mirabilis*	99.16%	LC432327.1
s9	*Halocynthia pyriformis* voucher	82.72%	MG422001.1	*Halocynthia igaboja*	99.64%	AY903925.1
s12	*Herdmania grandis*	82.28%	FJ528629.1	*Pyura dura*	97.54%	FM244856.1
s17	*Ascidiella aspersa*	100%	JQ742948.1	*Ascidiella* sp.	99.94%	FM244843.1
s20	*Ciona savignyi*	99.80%	JF919706.1	Uncultured eukaryote	99.38%	AB191429.1
s31	*Pyura haustor* voucher	81.70%	MH242956.1	*Pyura haustor*	99.82%	AY903926.1
s32	*Botryllus* sp. BT2	81.13%	LR745518.1	*Pelonaia corrugata*	97.92%	L12440.2
s37	*Ciona savignyi*	100%	JF919706.1	Uncultured eukaryote	99.76%	AB191429.1
s43	*Pyura haustor*	82.48%	MH242956.1	*Pyura haustor*	99.71%	AY903926.1
s44	*Costoanachis sparsa*	79.40%	KX422220.1	*Pyura haustor*	99.65%	AY903926.1
Z	*Ascidiella aspersa*	100%	KF309661.1	*Ascidiella* sp.	99.94%	FM244843.2

Suppl. Table 2 The best hit genes to the cloned genes in different ascidians.
In each individual, the best hit gene against Blastn (nr/nt) of COI and 18S is shown.

Suppl. Table3. Gene accession numbers used in the phylogenetic analysis

Species	COI	ref.	18S_1	ref.	18S_2	ref.
Ascidiella aspersa	NC_021469	[1]	KF699114	Direct Submission	AB610838	[2]
Ascidiella scabra	AB794965	[2]				
Botrylloides leachii	NC_024103	[3]	MG009583	[4]	JN573236	Direct Submission
Botryllus schlosseri	NC_021463	[5]	FM244858	[6]		
Branchiostoma floridae	NC_000834	[7]	M97571	[8]		
Ciona intestinalis	NC_017929	[9]	AB013017	[10]		
Ciona savignyi	NC_004570	[11]				
Halocynthia roretzi	AB024528	[12]	AB013016	[10]		
Herdmania momus	NC_013561	[13]	AF165827	[14]		
Molgula manhattensis	KU906100	Direct Submission	L12426	Direct Submission		
Phallusia mammilata	NC_009833	[15]	AF236803	[16]		
Phallusia fumigata	NC_009834	[17]	FM244844	[6]		
Styela clava	FJ528636.1	[18]	LC432329	[19]		
h1: *Ciona savignyi*	LC547008		LC547323			
h12: *Ascidia zara*	LC547009		LC547324			
h17: *Ascidia zara*	LC547010		LC547325			
m1: *Styela plicata*	LC546997		LC547313			
m3: *Microcosmus squamiger*	LC546998		LC547314			
m4: *Herdmania* sp.	LC546999		LC547315			
m5: *Polycarpa cryptocarpa*	LC547000		LC547316			
s1: Stolidobranchia sp. 1	LC547001					
s4: Stolidobranchia sp. 1	LC547002		LC547317			
s5: *Pyura* sp.	LC547003		LC547318			
s9: *Halocynthia hispida*	LC547004		LC547319			
s12: Stolidobranchia sp. 1	LC547005		LC547320			
s17: *Ascidiella aspersa*	LC547006		LC547321			
s20: *Ciona savignyi*	LC547011		LC547326			
s31: Stolidobranchia sp. 2	LC547012		LC547327			
s32: Stolidobranchia sp. 3	LC547013		LC547328			
s37: *Ciona savignyi*	LC547014		LC547329			
s43: Stolidobranchia sp. 4	LC547015		LC547330			
s44: Stolidobranchia sp. 4	LC547016		LC547331			
z: *Ascidiella aspersa*	LC547007		LC547322			

Suppl. Table 3 Gene accession numbers used in the phylogenetic analysis. From left column, we show the species names, accession number of COI, and accession number of 18S. For Ascidiella aspersa and Botrylloides leachii, the 18S sequences were short and in two different regions. The values in the 18S fragment sequences were assembled with GeneStudio.

references of **Suppl. Table 3**

1. Rubinstein ND, Feldstein T, Shenkar N, Botero-Castro F, Griggio F, Mastrototaro F, et al. Deep sequencing of mixed total DNA without barcodes allows efficient assembly of highly plastic Ascidian mitochondrial genomes. Genome Biol Evol. 2013;5:1185–99.
2. Nishikawa T, Oohara I, Saitoh K, Shigenobu Y, Hasegawa N, Kanamori M, et al. Molecular and Morphological Discrimination Between an Invasive Ascidian, Ascidiella aspersa , and Its Congener A. scabra (Urochordata: Ascidiacea). Zoolog Sci. 2014;31:180–5. doi:10.2108/zsj.31.180.
3. Griggio F, Voskoboynik A, Iannelli F, Justy F, Tilak MK, Xavier T, et al. Ascidian mitogenomics: Comparison of evolutionary rates in closely related taxa provides evidence of ongoing speciation events. Genome Biol Evol. 2014.
4. Reem E, Douek J, Rinkevich B. Ambiguities in the taxonomic assignment and species delineation of botryllid ascidians from the Israeli Mediterranean and other coastlines. Mitochondrial DNA Part A DNA Mapping, Seq Anal. 2018.
5. Voskoboynik A, Neff NF, Sahoo D, Newman AM, Pushkarev D, Koh W, et al. The genome sequence of the colonial chordate, Botryllus schlosseri. Elife. 2013.
6. Tsagkogeorga G, Turon X, Hopcroft RR, Tilak M-K, Feldstein T, Shenkar N, et al. An updated 18S rRNA phylogeny of tunicates based on mixture and secondary structure models. BMC Evol Biol. 2009;9:187. doi:10.1186/1471-2148-9-187.
7. Naylor GJ, Brown WM. Amphioxus mitochondrial DNA, chordate phylogeny, and the limits of inference based on comparisons of sequences. Syst Biol. 1998;47:61-76. doi: 10.1080/106351598261030.
8. Stock DW, Whitt GS. Evidence from 18S ribosomal RNA sequences that lampreys and hagfishes form a natural group. Science. 1992;257:787-9. doi: 10.1126/science.1496398.
9. Iannelli F, Pesole G, Sordino P, Gissi C. Mitogenomics reveals two cryptic species in Ciona intestinalis. Trends in Genetics. 2007.
10. Wada H. Evolutionary history of free-swimming and sessile lifestyles in urochordates as deduced from 18S rDNA molecular phylogeny. Mol Biol Evol. 1998.
11. Yokobori SI, Watanabe Y, Oshima T. Mitochondrial Genome of Ciona savignyi (Urochordata, Ascidiacea, Enterogona): Comparison of Gene Arrangement and tRNA Genes with Halocynthia roretzi Mitochondrial Genome. J Mol Evol. 2003.
12. Yokobori SI, Ueda T, Feldmaier-Fuchs G, Pääbo S, Ueshima R, Kondow A, et al. Complete DNA sequence of the mitochondrial genome of the ascidian Halocynthia roretzi (Chordata, Urochordata). Genetics. 1999.
13. Singh TR, Tsagkogeorga G, Delsuc F, Blanquart S, Shenkar N, Loya Y, et al. Tunicate mitogenomics and phylogenetics: Peculiarities of the Herdmania momus mitochondrial genome and support for the new chordate phylogeny. BMC Genomics. 2009.
14. Swalla BJ, Cameron CB, Corley LS, Garey JR. Urochordates are monophyletic within the deuterostomes. Syst Biol. 2000.
15. Kawamura K, Yoshida T, Sekida S. Autophagic dedifferentiation induced by cooperation between TOR inhibitor and retinoic acid signals in budding tunicates. Dev Biol. 2018;433:384–93. doi:10.1016/j.ydbio.2017.08.023.
16. Cameron CB, Garey JR, Swalla BJ. Evolution of the chordate body plan: New insights from phylogenetic analyses of deuterostome phyla. Proc Natl Acad Sci U S A. 2000.
17. Iannelli F, Griggio F, Pesole G, Gissi C. The mitochondrial genome of Phallusia mammillata and Phallusia fumigata (Tunicata, Ascidiacea): High genome plasticity at intra-genus level. BMC Evol Biol. 2007.
18. Pérez-Portela R, Bishop JDD, Davis AR, Turon X. Phylogeny of the families Pyuridae and Styelidae (Stolidobranchiata, Ascidiacea) inferred from mitochondrial and nuclear DNA sequences. Mol Phylogenet Evol. 2009.
19. Hasegawa N, Kajihara H. A redescription of syncarpa composita (Ascidiacea, stolidobranchia) with an inference of its phylogenetic position within styelidae. Zookeys. 2019;2019:1–15.

Suppl.Table 4 Diameters of eggs (n=10 each)

individual ID	diameter / μm
C: *Ciona robusta*	145.1 ± 1.8
h1: *Ascidia zara*	153.2 ± 2.7
h12: *Ascidia zara*	128.1 ± 1.7
h17: *Ascidia zara*	135 ± 2.1
m1: *Styela plicata*	143.8 ± 9.2
m3: *Microcosmus squamiger*	168.1 ± 7.1
m4: *Herdmania* sp.	236.5 ± 5.7
m5: *Polycarpa cryptocarpa*	214.0 ± 7.7
s1: Stolidobranchia sp. 1	371.4 ± 8.6
s4: Stolidobranchia sp. 1	368.0 ± 18.5
s5: *Pyura* sp.	120.0 ± 3.2
s9: *Halocynthia hispada*	209.0 ± 13.8
s12: Stolidobranchia sp. 1	381.4 ± 13.3
s17: *Ascidiella aspersa*	175.7 ± 2.6
s20: *Ciona savignyi*	157.8 ± 2.5
s31: Stolidobranchia sp. 2	192.3 ± 9.5
s32: Stolidobranchia sp. 3	138.5 ± 4.9
s37: *Ciona savignyi*	157.4 ± 2.2
s43: Stolidobranchia sp. 4	198.4 ± 6.3
s44: Stolidobranchia sp. 4	185.3 ± 7.3
Z: *Ascidiella aspersa*	175.4 ± 3.3

Suppl. Table 4 The egg diameters in different ascidian individuals.
Individual abbreviations are the same as in Fig. 1.

Suppl.Table5 bio-transparency of different ascidian species

Species	Transparency (%)	n (eggs) number
Z: *Ascidiella aspersa*	88.7 ± 0.7	6
s17: *Ascidiella aspersa*	88.0 ± 1.6	16
m5: *Polycarpa cryptocarpa*	60.0 ± 4.5	9
h17: *Ascidia zara*	50.7 ± 3.1	11
h12: *Ascidia zara*	48.9 ± 4.4	10
s5: *Pyura* sp.	44.6 ± 2.2	15
m3: *Microcosmus squamiger*	34.4 ± 2.7	7
m1: *Styella plicata*	31.6 ± 2.8	8
s20: *Ciona savignyi*	27.5 ± 0.8	10
s32: Stolidobranchia sp. 3	27.0 ± 2.7	9
m4: *Herdmania* sp.	22.1 ± 1.6	12
s44: Stolidobranchia sp. 4	20.4 ± 2.6	10
s37: *Ciona savignyi*	20.0 ± 1.0	9
h1: *Ciona savignyi*	19.8 ± 1.0	9
C: *Ciona robusta*	18.7 ± 1.2	14
s31: Stolidobranchia sp. 2	16.9 ± 1.2	9
s43: Stolidobranchia sp. 4	16.7 ± 1.1	10
s9: *Halocynthia hispida*	15.0 ± 0.68	9
s1: Stolidobranchia sp. 1	12.5 ± 0.3	5
s12: Stolidobranchia sp. 1	11.3 ± 0.6	6
s4: Stolidobranchia sp. 1	10.4 ± 0.4	5

Suppl. Table 5 List of egg bio-transparency values for the 21 individuals.
From left column, individual ID/species name, calculated value of bio-transparency (%), and number of egg samples.

Suppl.Fig. 1

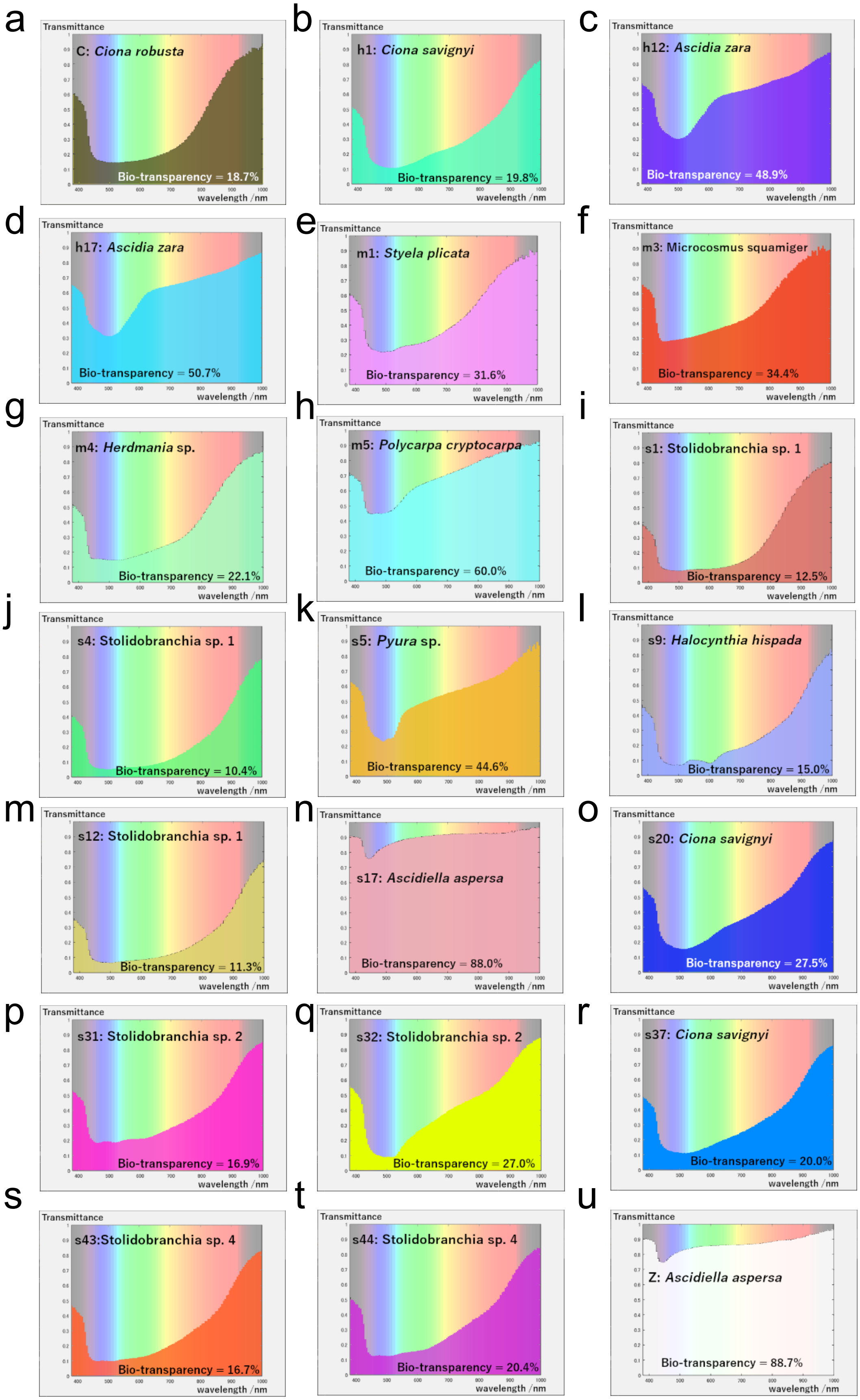

Suppl. Fig. 1 Transmittance (τ) of different ascidian eggs measured by the hyperspectral camera.

a cultured Ciona robusta individual at Misaki, b h1, c h12, and d h17 are three individuals from Honmoku; e m1, f m3, g m4, and h m5 are four individuals from Misaki; i s1, j s4, k s5, l s9, m s12, n s17, o s20, p s31, q s32, r s37, s s43, and t s44 are from Sado; and u Z from Onagawa.
